# Synthesis and Cytotoxic Evaluation of 3-Methylidenechroman-4-ones

**DOI:** 10.3390/molecules24101868

**Published:** 2019-05-15

**Authors:** Jacek Kędzia, Tomasz Bartosik, Joanna Drogosz, Anna Janecka, Urszula Krajewska, Tomasz Janecki

**Affiliations:** 1 Institute of Organic Chemistry, Lodz University of Technology, Żeromskiego 116, 90-924 Łódź, Poland; jacek.kedzia@p.lodz.pl (J.K.); tomasz.bartosik92@gmail.com (T.B.); 2 Department of Biomolecular Chemistry, Medical University of Łódź, Mazowiecka 6/8, 92-215 Łódź, Poland; joanna.drogosz@studumed.lodz.pl (J.D.); anna.janecka@umed.lodz.pl (A.J.); 3 Department of Pharmaceutical Biochemistry and Molecular Diagnostics, Faculty of Pharmacy, Medical University of Łódź, Muszyńskiego 1, 90-151 Łódź, Poland; ukrajewska@tlen.pl

**Keywords:** 3-methylidenechroman-4-ones, Michael addition, Horner–Wadsworth–Emmons olefination, cancer cell lines, apoptosis

## Abstract

In the search for new anticancer agents, a library of variously substituted 3-methylidenechroman-4-ones was synthesized using Horner–Wadsworth–Emmons methodology. Acylation of diethyl methylphosphonate with selected ethyl salicylates furnished 3-diethoxyphosphorylchromen-4-ones which were next used as Michael acceptors in the reaction with various Grignard reagents. The adducts were obtained as the mixtures of *trans* and *cis* diastereoisomers along with a small amount of enol forms. Their relative configuration and preferred conformation were established by NMR analysis. The adducts turned up to be effective Horner–Wadsworth–Emmons reagents giving 2-substituted 3-methylidenechroman-4-ones, which were then tested for their possible cytotoxic activity against two leukemia cell lines, HL-60 and NALM-6, and against MCF-7 breast cancer cell line. All new compounds (**14a**–**o**) were highly cytotoxic for the leukemic cells and showed a moderate or weak effect on MCF-7 cells. Analog **14d** exhibited the highest growth inhibitory activity and was more potent than carboplatin against HL-60 (IC_50_ = 1.46 ± 0.16 µM) and NALM-6 (IC_50_ = 0.50 ± 0.05 µM) cells. Further tests showed that **14d** induced apoptosis in NALM-6 cells, which was mediated mostly through the extrinsic pathway.

## 1. Introduction

**Figure 1:**
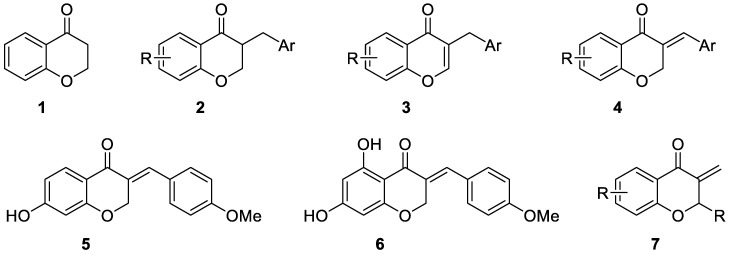
Structure and representative examples of homoisoflavonoids.

Chroman-4-one skeleton **1** is a core structure for a large group of plant metabolites called flavonoids, which possess many desirable biological activities including anticancer, antibacterial, and antioxidant properties [1,2]. One relatively small subgroup of flavonoids are homoisoflavonoids **2**–**4** which are characterized by the presence of arylmethyl or arylidene group in position 3 (Figure 1). A special place within this group belongs to 3-arylidenechroman-4-ones **4** which were found in many plants. For example, Bonducellin **5** was isolated from *Caesalpina bonducella* [3] and Eucomin **6** from Eucomis bicolor BAK (Liliaceae) [4].

Both natural and synthetic 3-arylidenechroman-4-ones **4** display valuable biological activities. They are potent and selective monoamine oxidase-B (MAO-B) inhibitors [5,6] and possess anti-cholinesterase activity [7,8], what makes them good candidates for the treatment of various neurological diseases such as Alzheimer’s or Parkinson’s disease. Furthermore, they show significant cytotoxicity for several cancer cell lines [9,10] and are cytochrome P450 aromatase inhibitors [11] being used for the treatment of advanced breast cancer. Also, their antifungal [12,13], antioxidant [14,15], and anti-inflammatory [16] activity was reported.

On the other hand, 3-methylidenechroman-4-ones **7**, which are structurally closely related to 3-arylidenechroman-4-ones **4**, have not been found in nature. Nevertheless, several syntheses of these compounds were reported. 2-Aryl-3-methylidenechroman-4-ones were obtained by Mannich reaction with 2-arylchroman-4-ones [17,18] and 2-alkyl(aryl)-3-methylidenechroman-4-ones were prepared by palladium-catalyzed tandem carbonylation-allene insertion reaction [19,20]. 3-Methylidenechroman-4-one was prepared by direct α-methylidenation mediated by diisopropylammonium trifluoroacetate [21] or by dehydration of 3-hydroxymethylchromen-4-one in the presence of methanesulfonylchloride [22]. Finally, 2-alkoxycarbonylmethyl-3-methylidenechroman-4-ones were obtained from enol silyl ethers of the corresponding chroman-4-ones [23,24]. Unfortunately, most of these methods have a very limited scope and/or are inefficient. For example, yields of the Mannich reactions were very low (3–22%) [17] or were not given because the obtained, crude 3-methylidenechroman-4-ones were used in further transformations [18]. On the other hand, palladium-catalyzed insertion reactions were more effective (23–90% yield) but required not readily available allenes as substrates. In turn the scope of direct α-methylidenation, dehydration of 3-hydroxymethylchromen-4-one or use the enol silyl ethers was limited to a single example in each case. 

In contrast to 3-arylidenechroman-4-ones **4**, biological activity of 3-methylidenechroman-4-ones **7** is poorly recognized. There is only one report describing their significant bacteriostatic activity against Gram-positive microorganisms [17]. However, an *exo*-cyclic methylidene bond conjugated with a carbonyl group present in 3-methylidenechroman-4-ones **7** is a structural motif found also in a large number of natural products, such as α-methylidene-γ- and δ-lactones [25,26] which react by the Michael-type addition with various bionucleophiles, disrupting key biological processes and are considered promising anticancer agents [27,28]. Consequently, we reasoned that also 3-methylidenechroman-4-ones **7** might have considerable cytotoxic activity.

In this report, we present a new, general synthetic method for obtaining variously substituted 3-methylidenechroman-4-ones **14**, based on the (well-recognized in our laboratory) Horner–Wadsworth–Emmons approach for the construction of *exo*-methylidene bond [29,30,31]. All obtained 3-methylidenechroman-4-ones **14** were evaluated in terms of their cytotoxic activity against three human cancer cell lines: promyelocytic leukemia HL-60, NALM-6, and breast adenocarcinoma cell line MCF-7. The most cytotoxic compound, **14d** was selected for further experiments and its effect on the induction of apoptosis was investigated. 

## 2. Results and Discussion

### 2.1. Chemistry

**Scheme 1:**
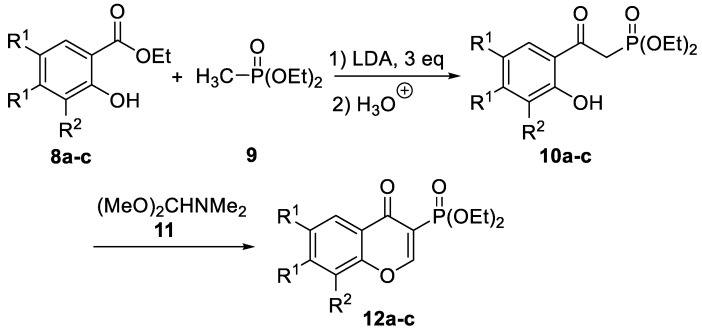
Synthesis of 3-diethoxyphosphorylchromen-4-ones **12a**–**c**.

The first step was the synthesis of 3-diethoxyphosphorylchromen-4-ones **12a**–**c**, which are crucial intermediates in our methodology. Literature search revealed that there is no efficient method for obtaining 2-unsubstituted 3-phosphorylchromen-4-ones. In the only report we have found, 3-diethoxyphosphorylchromen-4-one was formed in 5% yield, as a side product in free radical phosphorylation of chromen-4-one [32]. Therefore, we worked out a two-step procedure, which starts with the reaction of commercially available ethyl salicylates **8a**–**c** with diethyl methylphosphonate **9** in the presence of three equivalents of LDA (Scheme 1). Using this stoichiometry, protection of the hydroxyl group is avoided and we believe this is the major improvement over the reported acylation of dimethyl methylphosphonate using benzyl protected methyl salicylate [33]. The standard work-up and column chromatography purification gave 2-(2-hydroxyphenyl)-2-oxoethylphoshonates **10a**–**c** in good yields (Table 1). In the next step, reaction between phophonates **10a**–**c** and dimethylformamide dimethyl acetal **11** gave, after purification by column chromatography, 3-diethoxyphosphorylchromen-4-ones **12a**–**c** in high yields (Table 1 and Supplementary Materials).

**Scheme 2:**
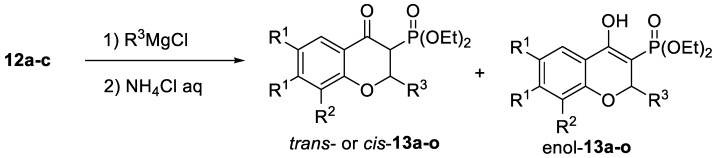
Synthesis of Michael adducts **13a**–**o**.

With 3-diethoxyphosphorylchromen-4-ones **12a**–**c** in hand, we performed their reactions with various Grignard reagents (Scheme 2). In all cases, after standard work-up, we received adducts **13a**–**o**, which were purified by column chromatography with yields given in Table 2. Interestingly, examination of the ^1^H, ^13^C and ^31^P NMR spectra revealed that all adducts **13** were formed as mixtures of *trans* and *cis* diastereoisomers (*trans-* or *cis-***13a**–**o**), along with small amount of enol form (enol-**13a**–**o**), with *trans* diastereoisomers strongly predominating. Ratios, determined from the ^31^P NMR spectra of the crude reaction mixtures, are given in Table 2. 

**Figure 2:**
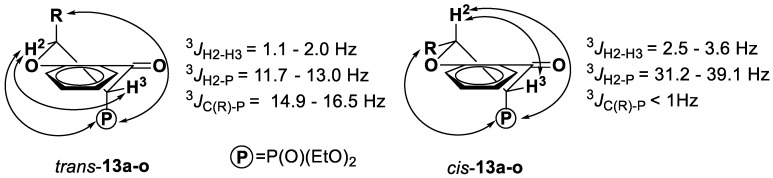
Half-chair conformation of *trans*- and *cis*-**13a**–**o** and characteristic ^3^*J*_H2-H3_, ^3^*J*_H2-P_ and ^3^*J*_C(R3)-P_ coupling constants.

Careful analysis of the NMR spectra showed also that both *trans*- and *cis*-**13a**–**o** exist in the half-chair conformation and diethoxyphosphoryl group occupies the axial position (Figure 2). Simple application of Karplus correlation between corresponding dihedral angles and coupling constants ^3^*J*_H2-H3_, ^3^*J*_H2-P_ and ^3^*J*_C(R3)-P_, determined from the ^1^H and ^13^C NMR spectra of *trans*- and *cis*-**13a**–**o** shows full agreement with the proposed configurations and conformations. Corresponding coupling constants are given in Figure 2. Similar half-chair conformation with diethoxyphosphoryl group in axial position was reported for 3-diethoxyphosphorylchroman-2-ones [34,35].

**Figure 3:**
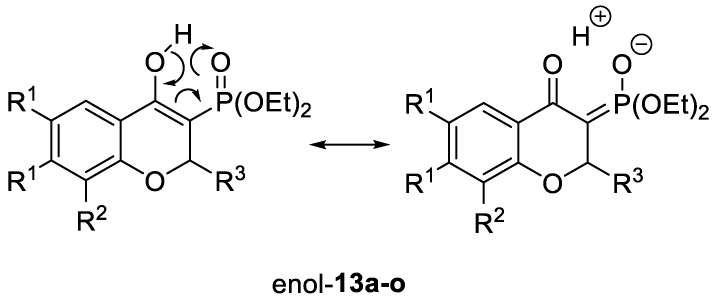
Two main resonance structures involved in the resonance-assisted hydrogen bond (RAHB) in 3-diethoxyphosphoryl-2*H*-chromen-4-oles.

On the other hand, ^1^H NMR spectra of enols-**13a**–**o** revealed very characteristic doublets with coupling constant ^4^*J*_P-H_ ~ 1 Hz and chemical shift in the range of 11–12 ppm, which can be assigned to the proton of the hydroxyl group. Coupling between this proton and phosphorus indicates the presence of resonance-assisted hydrogen bond (RAHB) [36] and can be visualized by resonance structures shown in Figure 3. Recently, we have reported on the existence of RAHB in 3-(dimenthoxyphosphoryl)-2-phenyl-1,2-dihydroquinolin-4-ol [37], what was the first example of this phenomenon in organophosphorus compounds. Now, we can confirm the existence of RAHB also in 3-diethoxyphosphoryl-2*H*-chromen-4-oles. 

**Scheme 3:**
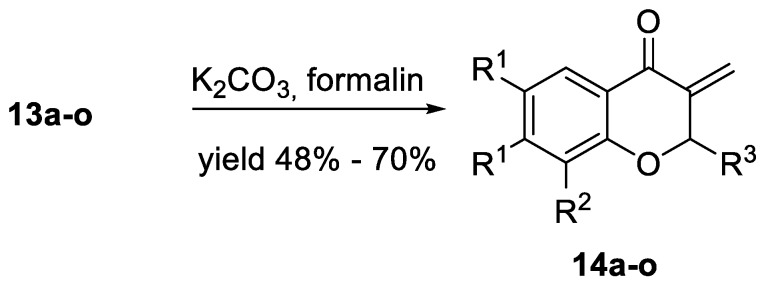
Synthesis of 3-methylidenechroman-4-ones **14a**–**o**.

Finally, all adducts **13a-o** were transformed into 3-methylidenechroman-4-ones **14a**–**o** performing Horner–Wadsworth–Emmons olefination of formaldehyde. The best results were obtained with K_2_CO_3_ used as a base and formalin as a source of formaldehyde (Scheme 3). Standard work-up and purification by column chromatography furnished 3-methylidenechroman-4-ones **14a**–**o** in moderate to good yields (Table 2).

### 2.2. Biology

#### 2.2.1. In Vitro Cytotoxicity of New Analogs Against Three Cancer Cell Lines

All obtained 3-methylidenechroman-4-ones **14a**–**o** were evaluated for their possible cytotoxic activity against three human cancer cell lines: leukemia HL-60 and NALM-6 and breast adenocarcinoma MCF-7 using the MTT assay (after 48 h incubation) (Table 3). Carboplatin served as a reference compound. 

Analysis of the structure–activity relationship revealed that chromanones **14a**–**j** were, in general, more potent than benzochromanones **14k**–**o**, containing additional benzene ring *ortho*-fused with a chromanone skeleton. All compounds (**14a**–**o**) were much more cytotoxic for leukemia cells than for the solid tumor MCF-7 cells. In both series of chromanones, **14a**–**e** (R^2^ = H) and **14f**–**j** (R^2^ = Me), the most potent compounds against leukemic HL-60 and NALM-6 cells were these containing an *i*-propyl substituent in position 2, i.e., **14d** and **14i**, respectively. For HL-60 cells only **14d** was more cytotoxic than the reference carboplatin. The highest cytotoxicity was observed against NALM-6 cells, with three analogs **14b**, **14d,** and **14i** exhibiting lower half maximal inhibitory concentration values (IC_50_) than carboplatin. Analog **14d** was the most cytotoxic chromanone for NALM-6 cells (IC_50_ of 0.5 ± 0.05 µM) and was selected for further investigation of its potential antineoplastic properties. 

#### 2.2.2. Apoptotic Cell Death Determination

**Figure 4:**
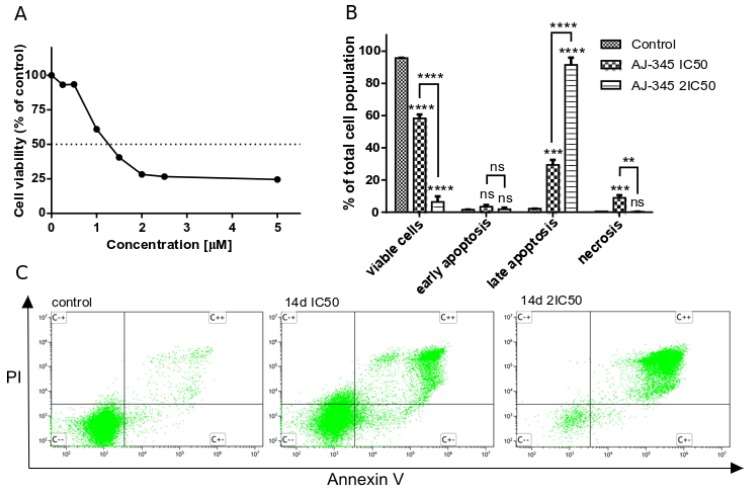
Effect of **14d** on induction of apoptosis in NALM-6 cells. (**A**) The cytotoxic activity of **14d** on NALM-6 cells after 24 h incubation; (**B**) Quantitative analysis of apoptosis by flow cytometry. The data are presented as mean ± SEM of three independent experiments. Statistical significance was determined using one-way ANOVA and a post-hoc multiple comparison Student–Newman–Keuls test. **** *p* < 0.0001; *** *p* < 0.001; ** *p* < 0.01; ns—not statistically significant. (**C**) Representative results of cell apoptosis obtained by Annexin V and PI staining using flow cytometry in NALM-6 cells untreated (control) or treated with **14d** at IC_50_ and 2 IC_50_ concentrations for 24 h.

It is now well documented that most anticancer drugs induce apoptosis. One of the main characteristics of apoptosis, phosphatidylserine (PS) translocation to the outer surface of the cellular membrane, was investigated by double-staining with Annexin V and propidium iodide (PI). Annexin V is a protein exerting high affinity to PS exposed on the outer surface of the plasma membrane, enabling detection of even an early stage apoptosis. The late stage of apoptosis is characterized by loss of membrane integrity allowing permeation of a dye such as PI into the cells [38,39]. Treatment of NALM-6 cells for 24 h (Figure 4A) with the selected analog **14d** at 1.25 µM (IC_50_) and 2.5 µM (2 IC_50_) concentrations led to the increase of Annexin V and PI-positive cells from 2.2% to 29.4% and 91.4%, respectively (Figure 4B,C), showing that **14d** induced the late stage of apoptosis in NALM-6 cells.

**Figure 5:**
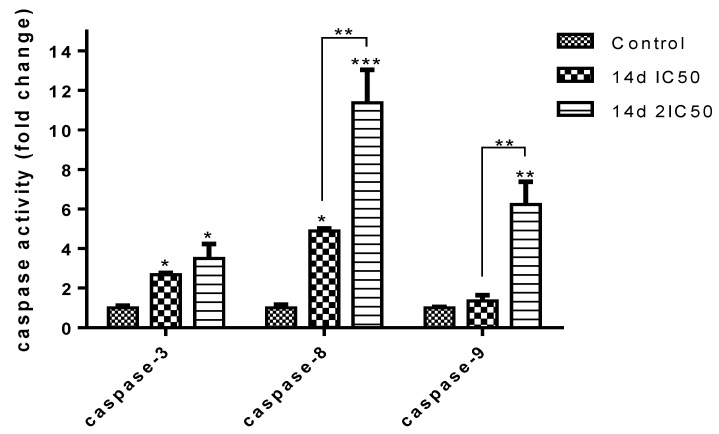
Activity of caspase 3, 8 and 9 in HL-60 cells after 6 h treatment with analog **14d** at 1.25 µM (IC_50_) and 2.5 µM (2IC_50_) concentrations. Results are expressed as mean ± SEM of triplicate experiment. Statistical significance was assessed using one-way ANOVA and a post-hoc multiple comparison Student–Newman–Keuls test; *** *p* < 0.001; ** *p* < 0.01; * *p* < 0.05.

Apoptosis occurs when caspases, which have proteolytic activity, cleave specific substrates, causing cell death. Depending on the initiator caspase involved in this process, the apoptosis may be mediated by the intrinsic (caspase 9) or extrinsic (caspase 8) pathway [40]. To investigate which caspases were involved in the apoptosis inducted by analog **14d** in NALM-6 cells, the cells were treated with **14d** at 1.25 µM and 2.5 µM concentrations for 6 h. Then, the activity of executioner caspase 3 and initiator caspases 8 and 9 was quantified using fluorogenic indicators. Results presented in Figure 5 indicate that the levels of caspase 3, 8 and 9 were significantly increased: 2.7- and 3.7-fold for caspase 3, 4.9- and 11.3-fold for caspase 8, and 1.25- and 5.7-fold for caspase 9 after treatment of the cells with 1.25 µM and 2.5 µM concentration of **14d**, respectively. Activation of the extrinsic pathway was more prominent than the intrinsic pathway. 

Presented results indicate that compound **14d** is a potent cytotoxic agent that significantly inhibits metabolic activity of NALM-6 cells with IC_50_ value as low as 0.5 µM. Analog **14d** also promotes apoptosis in the investigated cell line, which is mediated by the extrinsic and in a much lesser extend intrinsic pathway. 

## 3. Materials and Methods

### 3.1. Chemistry 

#### 3.1.1. General Information

NMR spectra were recorded on a Bruker DPX 250 or Bruker Avance II instrument at 250.13 MHz or 700 MHz for ^1^H, 62.9 MHz or 176 MHz for ^13^C, and 101.3 MHz for ^31^P NMR with tetramethylsilane used as an internal and 85% H_3_PO_4_ as an external standard. ^31^P NMR spectra were recorded using broadband proton decoupling. IR spectra were recorded on a Bruker Alpha ATR spectrophotometer. Melting points were determined in open capillaries and are uncorrected. Optical rotations were measured on a Perkin-Elmer 241 polarimeter. The [α]_D_ values are given in deg·cm2·g^−1^ and concentration c in g·(100 mL)^−1^. Column chromatography was performed on silica gel 60 (230–400 mesh) (Aldrich, Steinheim, Germany). Thin-layer chromatography was performed on the pre-coated TLC sheets of silica gel 60 F254 (Aldrich, Steinheim, Germany). The purity of the synthesized compounds was confirmed by the combustion elemental analyses (CHN, elemental analyzer EuroVector 3018, Elementar Analysensysteme GmbH (Langenselbold, Germany). MS spectra were recorded on Waters 2695-Waters ZQ 2000 LC/MS apparatus (Waters Corporation, Milford, MA, USA). All reagents and starting materials were purchased from commercial vendors and used without further purification. Organic solvents were dried and distilled prior to use. Standard syringe techniques were used for transferring dry solvents.

#### 3.1.2. General Procedure for the Synthesis of 2-Substituted 3-Metylidenechroman-4-ones **14a**–**o**.

To the vigorously stirred solution of 2-substituted 3-diethoxychroman-4-on **13a**–**o** (0.15 mmol) in THF (1.5 mL), formaldehyde (36–38% solution in water, 0.125 mL, ca. 1.50 mmol) was added at 0 °C, followed by addition of K_2_CO_3_ (41 mg, 0.30 mmol) in water (0.4 mL). The resulting mixture was stirred vigorously at 0 °C for 3 h. Next Et_2_O (5 mL) was added and layers were separated. The water fraction was washed with Et_2_O (5 mL). Organic fractions were combined, washed with brine (5 mL) and dried over MgSO_4_. The solvents were evaporated under reduced pressure and the resulting crude product was then purified by column chromatography (eluent CH_2_Cl_2_).

*2-Methyl-3-methylidenechroman-4-one* (**14a**) (17.7 mg, 68%). Colourless oil. ^1^H NMR (700 MHz, Chloroform-*d*) δ 1.63 (d, *J* = 6.4 Hz, 3H), 5.03–5.14 (m, 1H), 5.55 (dd, *J* = 2.0, 0.8 Hz, 1H), 6.32 (dd, *J* = 1.6, 0.8 Hz, 1H), 6.95 (dd, *J* = 8.3, 1.0 Hz, 1H), 7.03 (ddd, *J* = 8.1, 7.2, 1.1 Hz, 1H), 7.47 (ddd, *J* = 8.6, 7.1, 1.8 Hz, 1H), 7.97 (dd, *J* = 7.9, 1.8 Hz, 1H). ^13^C NMR (176 MHz, Chloroform-*d*) δ 19.03, 76.36, 118.30, 121.44, 121.59, 121.82, 128.00, 136.14, 143.68, 161.30, 182.66. ESI-MS [M + H]^+^ = 175.2. Anal. Calcd for C_11_H_10_O_2_: C, 75.84%; H, 5.79%. Found: C, 75.61%, H, 5.71%.

*2-Ethyl-3-methylidenechroman-4-one* (**14b**) (20.1 mg, 71%). Colourless oil. ^1^H NMR (700 MHz, Chloroform-*d*) δ 1.04 (t, *J* = 7.3 Hz, 3H), 1.75–1.89 (m, 1H), 1.91–2.03 (m, 1H), 4.90 (t, *J* = 7.0 Hz, 1H), 5.50 (s, 1H), 6.33 (s, 1H), 6.96 (d, *J* = 8.4 Hz, 1H), 7.02 (t, *J* = 7.5 Hz, 1H), 7.47 (s, 1H), 7.95 (d, *J* = 7.9 Hz, 1H). ^13^C NMR (176 MHz, Chloroform-*d*) δ 9.83, 26.65, 81.89, 118.47, 121.41, 121.66, 122.33, 127.84, 136.21, 142.36, 160.77, 182.49. ESI-MS [M + H]^+^ = 189.3. Anal. Calcd for C_12_H_12_O_2_: C, 76.57%; H, 6.43%. Found: C, 76.41%, H, 6.51%.

*2-Butyl-3-methylidenechroman-4-one* (**14c**) (19.1 mg, 59%). Colourless oil. ^1^H NMR (700 MHz, Chloroform-*d*) δ 0.91 (t, *J* = 7.3 Hz, 3H), 1.31–1.45 (m, 3H), 1.47–1.55 (m, 1H), 1.71–1.80 (m, 1H), 1.89–2.00 (m, 1H), 4.86–5.10 (m, 1H), 5.50 (t, *J* = 1.2 Hz, 1H), 6.32 (t, *J* = 1.0 Hz, 1H), 6.95 (dd, *J* = 8.4, 1.0 Hz, 1H), 7.02 (ddd, *J* = 8.1, 7.2, 1.1 Hz, 1H), 7.47 (ddd, *J* = 8.6, 7.1, 1.8 Hz, 1H), 7.96 (dd, *J* = 7.8, 1.8 Hz, 1H).^13^C NMR (176 MHz, Chloroform-*d*) δ 14.09, 22.48, 27.50, 33.18, 80.64, 118.48, 121.43, 121.66, 122.17, 127.85, 136.22, 142.65, 160.78, 182.56. ESI-MS [M + H]^+^ = 217.4. Anal. Calcd for C_14_H_16_O_2_: C, 77.75%; H, 7.46%. Found: C, 77.52%, H, 7.25%. 

*2-Isopropyl-3-methylidenechroman-4-one* (**14d**) (16.1 mg, 53%). Colourless oil. ^1^H NMR (700 MHz, Chloroform-*d*) δ 0.90 (d, *J* = 6.8 Hz, 3H), 1.04 (d, *J* = 6.6 Hz, 3H), 1.96–2.16 (m, 1H), 4.62 (dt, *J* = 8.5, 1.1 Hz, 1H), 5.46 (t, *J* = 1.2 Hz, 1H), 6.36 (t, *J* = 1.0 Hz, 1H), 6.95 (ddd, *J* = 8.3, 1.1, 0.5 Hz, 1H), 7.00 (ddd, *J* = 8.0, 7.1, 1.1 Hz, 1H), 7.47 (ddd, *J* = 8.3, 7.1, 1.8 Hz, 1H), 7.93 (ddd, *J* = 7.9, 1.8, 0.5 Hz, 1H). ^13^C NMR (176 MHz, Chloroform-*d*) δ 18.51, 18.83, 30.78, 86.66, 118.41, 121.49, 121.57, 123.80, 127.66, 136.31, 141.26, 160.53, 182.44. ESI-MS [M + H]^+^ = 203.0. Anal. Calcd for C_13_H_14_O_2_: C, 77.20%; H, 6.98%. Found: C, 77.42%, H, 7.05%.

*3-Methylidene-2-phenylchroman-4-one* (**14e**) (29.8 mg, 84%). Yelowish oil. ^1^H NMR (700 MHz, Chloroform-*d*) δ 5.16–5.23 (m, 1H), 6.02 (d, *J* = 1.7 Hz, 1H), 6.44 (t, *J* = 1.2 Hz, 1H), 7.35–7.39 (m, 1H), 7.39–7.45 (m, 4H), 7.50 (ddd, *J* = 8.7, 7.2, 1.8 Hz, 1H), 7.99 (dd, *J* = 7.9, 1.7 Hz, 1H). ^13^C NMR (176 MHz, Chloroform-*d*) δ 82.49, 118.47, 121.76, 122.13, 125.09, 127.58, 128.02, 128.78, 128.87, 136.30, 137.27, 142.76, 161.14, 182.22. ESI-MS [M + H]^+^ = 237.4. Anal. Calcd for C_16_H_12_O_2_: C, 81.34%; H, 5.12%. Found: C, 81.42%, H, 5.23%.

*2,8-Dimethyl-3-methylidenechroman-4-one* (**14f**) (18.1 mg, 64%). Yelowish oil. ^1^H NMR (700 MHz, Chloroform-*d*) δ 1.64 (d, *J* = 6.5 Hz, 3H), 2.23 (s, 3H), 5.10 (qt, *J* = 6.4, 1.8 Hz, 1H), 5.54 (dd, *J* = 2.0, 0.9 Hz, 1H), 6.31 (dd, *J* = 1.6, 0.9 Hz, 1H), 6.93 (t, *J* = 7.6 Hz, 1H), 7.33 (ddd, *J* = 7.2, 1.8, 1.0 Hz, 1H), 7.82 (dd, *J* = 7.9, 1.7 Hz, 1H). ^13^C NMR (176 MHz, Chloroform-*d*) δ 15.72, 19.13, 76.19, 121.08, 121.21 × 2, 125.56, 127.50, 136.91, 143.77, 159.53, 183.04. ESI-MS [M + H]^+^ = 189.0. Anal. Calcd for C_12_H_12_O_2_: C, 76.57%; H, 6.43%. Found: C, 76.42%, H, 6.55%.

*2-Ethyl-8-methyl-3-methylidenechroman-4-one* (**14g**) (17.9 mg, 59%). Yelowish oil. ^1^H NMR (700 MHz, Chloroform-*d*) δ 1.07 (t, *J* = 7.4 Hz, 3H), 1.73 – 1.86 (m, 1H), 1.90–2.03 (m, 1H), 2.25 (t, *J* = 0.7 Hz, 3H), 4.76 – 5.05 (m, 1H), 5.50 (dd, *J* = 1.5, 1.0 Hz, 1H), 6.32 (t, *J* = 1.1 Hz, 1H), 6.92 (t, *J* = 7.5 Hz, 1H), 7.34 (ddd, *J* = 7.2, 1.8, 0.9 Hz, 1H), 7.81 (ddd, *J* = 7.9, 1.8, 0.7 Hz, 1H). ^13^C NMR (176 MHz, Chloroform-*d*) δ 10.09, 15.69, 26.69, 81.76, 121.08, 121.11, 121.88, 125.45, 127.66, 136.99, 142.60, 158.94, 182.87. ESI-MS [M + H]^+^ = 203.0. Anal. Calcd for C_13_H_14_O_2_: C, 77.20%; H, 6.98%. Found: C, 77.38%, H, 7.09%.

*2-Butyl-8-methyl-3-methylidenechroman-4-one* (**14h**) (20.0 mg, 52%). Yelowish oil. ^1^H NMR (700 MHz, Chloroform-*d*) δ 0.92 (t, *J* = 7.3 Hz, 3H), 1.29–1.49 (m, 3H), 1.49–1.58 (m, 1H), 1.71–1.80 (m, 1H), 1.89–2.01 (m, 1H), 2.24 (s, 3H), 4.98 (ddt, *J* = 9.0, 5.1, 1.4 Hz, 1H), 5.50 (t, *J* = 1.2 Hz, 1H), 6.30 (d, *J* = 1.2 Hz, 1H), 6.92 (t, *J* = 7.6 Hz, 1H), 7.31–7.39 (m, 1H), 7.81 (dd, *J* = 7.8, 1.7 Hz, 1H). ^13^C NMR (176 MHz, Chloroform-*d*) δ 14.12, 15.73, 22.44, 27.70, 33.12, 80.39, 121.12, 121.73, 125.46, 127.66, 136.99, 142.85, 158.99, 182.96. ESI-MS [M + H]^+^ = 231.0. Anal. Calcd for C_15_H_18_O_2_: C, 78.23%; H, 7.88%. Found: C, 78.44%, H, 8.05%.

*2-Isopropyl-8-methyl-3-methylidenechroman-4-one* (**14i**) (17.2 mg, 53%). Yelowish oil. ^1^H NMR (700 MHz, Chloroform-*d*) δ 0.92 (d, *J* = 6.7 Hz, 3H), 1.05 (d, *J* = 6.6 Hz, 3H), 1.95–2.12 (m, 1H), 2.26 (s, 3H), 4.66 (dd, *J* = 8.3, 1.3 Hz, 1H), 5.46 (d, *J* = 1.1 Hz, 1H), 6.35 (t, *J* = 1.1 Hz, 1H), 6.91 (t, *J* = 7.6 Hz, 1H), 7.34 (ddd, *J* = 7.2, 1.8, 0.9 Hz, 1H), 7.79 (dd, *J* = 7.9, 1.7 Hz, 1H). ^13^C NMR (176 MHz, Chloroform-*d*) δ 15.71, 18.72, 19.03, 30.58, 86.60, 120.97, 121.17, 123.42, 125.32, 127.48, 137.14, 141.40, 158.69, 182.86. ESI-MS [M + H]^+^ = 217.0. Anal. Calcd for C_14_H_16_O_2_: C, 77.75%; H, 7.46%. Found: C, 77.52%, H, 7.27%.

*8-Methyl-3-methylidene-2-phenylchroman-4-one* (**14j**) (22.1 mg, 59%). Yelowish oil. ^1^H NMR (700 MHz, Chloroform-*d*) δ 2.28 (d, *J* = 0.8 Hz, 3H), 5.23 (dd, *J* = 1.8, 1.1 Hz, 1H), 6.05 (t, *J* = 1.7 Hz, 1H), 6.42 (t, *J* = 1.3 Hz, 1H), 6.95 (t, *J* = 7.6 Hz, 1H), 7.33 – 7.38 (m, 2H), 7.38–7.45 (m, 4H), 7.83 (ddd, *J* = 7.9, 1.7, 0.7 Hz, 1H). ^13^C NMR (176 MHz, Chloroform-*d*) δ 15.86, 82.14, 121.45, 121.55, 124.67, 125.61, 127.31, 127.62, 128.71, 128.74, 137.16, 137.57, 142.76, 159.29, 182.71. ESI-MS [M + H]^+^ = 251.0. Anal. Calcd for C_17_H_14_O_2_: C, 81.58%; H, 5.64%. Found: C, 81.72%, H, 6.75%.

*2-Methyl-3-methylidene-2,3-dihydro-4H-benzo[g]chromen-4-one* (**14k**) (22.2 mg, 66%). Yelowish oil. ^1^H NMR (700 MHz, Chloroform-*d*) δ 1.65 (dd, *J* = 6.5, 1.2 Hz, 4H), 5.08–5.19 (m, 1H), 5.61 (dt, *J* = 1.7, 1.1 Hz, 1H), 6.41 (dt, *J* = 1.5, 0.7 Hz, 1H), 7.33 (d, *J* = 1.5 Hz, 1H), 7.36 (ddd, *J* = 8.1, 6.8, 1.2 Hz, 1H), 7.50 (ddt, *J* = 8.2, 6.8, 1.4 Hz, 1H), 7.70 (dd, *J* = 8.4, 1.5 Hz, 1H), 7.89 (dd, *J* = 8.2, 1.6 Hz, 1H), 8.58 (s, 1H). ^13^C NMR (176 MHz, Chloroform-*d*) δ 19.26, 76.01, 113.14, 121.83, 122.30, 124.74, 126.68, 128.80, 129.23, 129.99, 130.14, 137.88, 143.99, 156.15, 183.18. ESI-MS [M + H]^+^ = 255.0. Anal. Calcd for C_15_H_12_O_2_: C, 80.34%; H, 5.39%. Found: C, 80.22%, H, 5.27%.

*2-Ethyl-3-methylidene-2,3-dihydro-4H-benzo[g]chromen-4-one* (**14l**) (23.9 mg, 67%). Yelowish oil. ^1^H NMR (700 MHz, Chloroform-*d*) δ 1.08 (t, *J* = 7.4 Hz, 3H), 1.81 (dtd, *J* = 14.6, 7.5, 1.8 Hz, 1H), 1.97 (dtd, *J* = 14.2, 7.2, 1.2 Hz, 1H), 4.93 (ddt, *J* = 8.2, 5.6, 1.2 Hz, 1H), 5.57 (t, *J* = 1.1 Hz, 1H), 6.42 (d, *J* = 1.0 Hz, 1H), 7.34 (s, 1H), 7.36 (ddd, *J* = 8.2, 6.8, 1.2 Hz, 1H), 7.51 (ddd, *J* = 8.2, 6.7, 1.2 Hz, 1H), 7.71 (d, *J* = 8.4 Hz, 1H), 7.89 (d, *J* = 8.3 Hz, 1H), 8.57 (s, 1H). ^13^C NMR (176 MHz, Chloroform-*d*) δ 9.97, 27.15, 81.74, 113.48, 122.54, 122.75, 124.83, 126.73, 128.89, 129.35, 130.10, 130.15, 138.11, 142.84, 155.70, 183.18. ESI-MS [M + H]^+^ = 239.2. Anal. Calcd for C_16_H_14_O_2_: C, 80.65%; H, 5.92%. Found: C, 80.43%, H, 6.05%.

*2-Butyl-3-methylidene-2,3-dihydro-4H-benzo[g]chromen-4-one* (**14m**) (24.0 mg, 60%). Yelowish oil. ^1^H NMR (700 MHz, Chloroform-*d*) δ 1.36 (tdd, *J* = 15.8, 8.2, 4.4 Hz, 2H), 1.45 (dddd, *J* = 13.4, 9.6, 6.6, 4.0 Hz, 1H), 1.51 – 1.59 (m, 2H), 1.75 (ddt, *J* = 14.0, 10.7, 5.6 Hz, 1H), 1.92–2.00 (m, 1H), 5.00 (dd, *J* = 8.6, 5.6 Hz, 1H), 5.56 (d, *J* = 1.3 Hz, 1H), 6.40 (s, 1H), 7.34 (s, 1H), 7.36 (ddd, *J* = 8.1, 6.8, 1.1 Hz, 1H), 7.51 (ddd, *J* = 8.2, 6.8, 1.2 Hz, 1H), 7.71 (d, *J* = 8.4 Hz, 1H), 7.90 (d, *J* = 8.3 Hz, 1H), 8.58 (s, 1H). ^13^C NMR (176 MHz, Chloroform-*d*) δ 14.11, 22.49, 27.62, 33.69, 80.46, 113.49, 122.55, 122.58, 124.83, 126.75, 128.89, 129.35, 130.11, 130.17, 138.13, 143.11, 155.72, 183.26. ESI-MS [M + H]^+^ = 267.2. Anal. Calcd for C_18_H_18_O_2_: C, 81.17%; H, 6.81%. Found: C, 81.32%, H, 6.95%.

*2-Isopropyl-3-methylidene-2,3-dihydro-4H-benzo[g]chromen-4-one* (**14n**) (18.2 mg, 48%). Yelowish oil. ^1^H NMR (700 MHz, Chloroform-*d*) δ 0.91 (d, *J* = 6.7 Hz, 3H), 1.08 (d, *J* = 6.4 Hz, 3H), 1.97–2.06 (m, 1H), 4.62 (dd, *J* = 9.0, 0.9 Hz, 1H), 5.53 (t, *J* = 1.0 Hz, 1H), 6.45 (q, *J* = 0.9 Hz, 1H), 7.33 (s, 1H), 7.36 (ddt, *J* = 7.7, 6.7, 1.0 Hz, 1H), 7.51 (ddt, *J* = 7.7, 6.8, 1.0 Hz, 1H), 7.71 (d, *J* = 8.3 Hz, 1H), 7.89 (d, *J* = 8.3 Hz, 1H), 8.56 (s, 1H). ^13^C NMR (176 MHz, Chloroform-*d*) δ 18.64, 18.92, 31.11, 86.57, 113.41, 122.73, 124.26, 124.78, 126.70, 128.79, 129.34, 129.93, 130.16, 138.19, 141.67, 155.47, 183.19. ESI-MS [M + H]^+^ = 253.2. Anal. Calcd for C_17_H_16_O_2_: C, 80.93%; H, 6.39%. Found: C, 81.02%, H, 6.29%.

*3-Methylidene-2-phenyl-2,3-dihydro-4H-benzo[g]chromen-4-one* (**14o**) (30.1 mg, 70%). Yelowish oil. ^1^H NMR (700 MHz, Chloroform-*d*) δ 5.32 (dd, *J* = 1.7, 0.9 Hz, 1H), 6.07 (d, *J* = 1.7 Hz, 1H), 6.54 (t, *J* = 1.2 Hz, 1H), 7.36 (ddd, *J* = 7.4, 3.4, 2.1 Hz, 2H), 7.38 – 7.41 (m, 4H), 7.44–7.48 (m, 2H), 7.51 (ddd, *J* = 8.2, 6.7, 1.2 Hz, 1H), 7.71 (d, *J* = 8.3 Hz, 1H), 7.89 (d, *J* = 8.3 Hz, 1H), 8.59 (s, 1H). ^13^C NMR (176 MHz, Chloroform-*d*) δ 82.20, 113.65, 122.64, 125.00, 125.42, 126.88, 127.62, 128.82×2, 129.05, 129.44, 130.16, 130.36, 137.62, 138.05, 143.06, 156.14, 183.00. ESI-MS [M + H]^+^ = 287.2. Anal. Calcd for C_20_H_14_O_2_: C, 83.90%; H, 4.93%. Found: C, 83.72%, H, 5.05%.

### 3.2. Biology

#### 3.2.1. Cell Lines

HL-60, NALM-6, MCF-7 cell lines were purchased from the European Collection of Cell Cultures (ECACC). HL-60 and NALM-6 cells were cultured in RPMI 1640 Glutamax medium (Gibco/Life Technologies, Carlsbad, CA, USA), supplemented with 10% fetal bovine serum, penicillin and streptomycin. MCF-7 cells were maintained in EMEM growth medium (Sigma-Aldrich, St. Louis, MO, USA), supplemented with 2 mM glutamine, 10% fetal bovine serum, 1% NEAA and gentamycin.

#### 3.2.2. In Vitro Cytotoxicity Assay

The MTT (3-(4,5-dimethyldiazol-2-yl)-2,5 diphenyl tetrazolium bromide) assay was used to investigate the cytotoxicity of new analogs in HL-60, NALM-6 and MCF-7 cell lines. Briefly, cells were seeded in a 24-well plate at a concentration of 8 × 10^4^ cells/mL. Following initial incubation (20 h; 37 °C), cells were treated with various concentrations of the analogs for 48 h. Then, cells were incubated for 1.5 h with MTT solution (100 µL/well; 5 mg/mL of PBS). The plates were centrifuged (3000 rpm, 5 min.) and the supernatant was discarded. The formazan product was dissolved by addition of DMSO (1 mL/well). The absorbance was measured using FlexStation 3 Multi-Mode Microplate Reader (Molecular Devices, LLC) at 560 nm. Assay was performed in triplicate. Cell viability rate was calculated by dividing mean sample absorbance by mean control absorbance.

#### 3.2.3. Annexin V and Propidium Iodide Assay

Apoptotic cell death was determined using FITC Annexin V Apoptosis Detection Kit I (BD Biosciences, San Jose, CA, USA) in accordance with the manufacturer’s protocol. NALM-6 cells were seeded in 6-well plates at a density of 4.0 × 10^5^ cells/mL in 2 mL of cell culture medium. Cells were treated with 14d at IC_50_ and 2 IC_50_ concentrations (1.25 µM and 2.5 µM, respectively) for 24 h, at 37 °C. Untreated cells were used as a control. Then, cells were washed with PBS, resuspended in the binding buffer and stained with FITC Annexin V and propidium iodide (PI), followed by incubation in the dark at RT for 15 min. Finally, flow cytometry analysis of the cells was performed using CytoFLEX (Beckman Coulter, Inc., Brea, CA, USA). Data were analyzed using CytExpert Software (2.3, Beckman Coulter, Inc, Brea, CA, USA).

#### 3.2.4. Caspase 3, Caspase 8, and Caspase 9 Activity

Activity of the key caspases involved in apoptosis was simultaneously quantified using a fluorometric Caspase 3, Caspase 8 and Caspase 9 Multiplex Activity Assay Kit (Abcam, Cambridge, UK), according to the manufacturer’s protocol. Briefly, NALM-6 cells were seeded in a 96-well plate at a density of 2.0 × 10^5^ cells/90 µl of cell culture medium per well. The plates were centrifuged at 800 rpm for 2 min. and the cells were treated with **14d** at 1.25 µM and 2.5 µM concentrations. Medium without cells and untreated cells were used as controls. The cell plate was incubated for 6 h, 37 °C, 5% CO_2_. Caspase-9, -3, and -8 substrates were added to each well and the cell plate was incubated for 60 min. at RT, protected from light. The plate was centrifuged at 800 rpm for 2 min., followed by a fluorescence readout using FlexStation 3 Multi-Mode Microplate Reader (Molecular Devices, LLC) at specific wavelengths. The assay was performed in triplicate; blank readings were subtracted from all measurements and the fold change of fluorescent intensity between control and the treated cells was calculated. 

## 4. Conclusions

A simple and efficient synthesis of 3-methylidenechroman-4-ones **14a**–**o**, applying Horner–Wadsworth–Emmons methodology was described. Furthermore, relative configuration of the crucial intermediates for this methodology, trans- or cis- 2-substituted 3-diethoxyphosphorylchroman-4-ones **13a**–**o**, was elaborated using NMR analysis. The obtained library of 3-methylidenechroman-4-ones **14a**–**o** was evaluated for the anti-proliferative activity against leukemia and breast cancer cell lines. Several members of this library were determined to be capable of killing leukemia cells with improved activity compared to carboplatin used as a reference compound, exhibiting IC_50_ values in the low micromolar range. The most potent 2-isopropyl-3-methylidenechroman-4-one **14d** was then evaluated for its possible apoptotic activity against NALM-6 cells. Compound **14d** promoted apoptosis mediated by caspase 3/8 and in a much lesser extend by caspase 3/9 induction, which indicated that mostly the extrinsic pathway was engaged in the programmed cell death caused by this analog. These results show that compound **14d** is a good lead in a search for new anticancer agents.

## Figures and Tables

**Table 1:** Yields of phosphonates **10a-c** and 3-diethoxyphosphorylchromen-4-ones **12a**–**c**.

Compound	R^1^,R^1^	R^2^	10 Yield [%] ^1^	12 Yield [%] ^1^
**a**	H,H	H	80	92
**b**	H,H	Me	75	89
**c**	CH=CH-CH=CH	H	80	78

^1^ Yield of pure, isolated product, based on **8** or **10**, respectively.

**Table 2:** Adducts **13a-o** and methylidenechroman-4-ones **14a**–**o** obtained.

Compound	R^1^,R^1^	R^2^	R^3^	13		14 Yield [%] ^2^
				*trans*/*cis*/enol ^1^	Yield [%] ^2^	
**a**	H,H	H	Me	67/30/3	84	68
**b**	H,H	H	Et	71/25/4	68	71
**c**	H,H	H	*n*-Bu	76/20/4	85	59
**d**	H,H	H	iPr	71/26/3	66	53
**e**	H,H	H	Ph	81/10/9	64	84
**f**	H,H	Me	Me	73/24/3	74	64
**g**	H,H	Me	Et	75/22/3	76	59
**h**	H,H	Me	*n*-Bu	72/25/3	83	52
**i**	H,H	Me	iPr	72/26/2	70	53
**j**	H,H	Me	Ph	83/11/6	84	59
**k**	CH=CH-CH=CH	H	Me	79/14/7	81	66
**l**	CH=CH-CH=CH	H	Et	89/4/7	64	67
**m**	CH=CH-CH=CH	H	*n*-Bu	73/16/11	53	60
**n**	CH=CH-CH=CH	H	iPr	81/15/4	66	48
**o**	CH=CH-CH=CH	H	Ph	65/6/29	76	70

^1^ Ratios taken from ^31^P NMR spectra of the crude mixtures. ^2^ Yield of pure, isolated product, based on **12** or **13**, respectively.

**Table 3:** Tumor cell growth inhibitory activity of **14a**–**o** on three cancer cell lines.

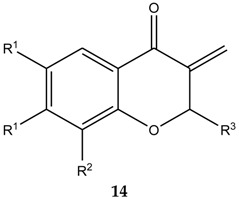

14	R^1^, R^1^	R^2^	R^3^	IC_50_ [µM] ^1^		
				HL-60	NALM-6	MCF-7
**a**	H, H	H	Me	5.91 ± 0.32	2.13 ± 0.04	9.70 ± 0.80
**b**	H, H	H	Et	5.24 ± 0.52	0.60 ± 0.02	12.50 ± 0.71
**c**	H, H	H	*n*-Bu	8.79 ± 0.81	4.23 ± 0.46	16.00 ± 0.20
**d**	H, H	H	*i*Pr	1.46 ± 0.16	0.50 ± 0.05	19.40 ± 0.80
**e**	H, H	H	Ph	23.86 ± 2.30	5,76 ± 0.23	17,10 ± 0.30
**f**	H, H	Me	Me	7.52 ± 0.81	3.28 ± 0.35	10.50 ± 0.34
**g**	H, H	Me	Et	20.87 ± 1.73	4.83 ± 0.45	11.60 ± 0.07
**h**	H, H	Me	*n*-Bu	32.16 ± 3.17	6.36 ± 0.36	13.50 ± 0.60
**i**	H, H	Me	*i*Pr	3.45 ± 0.34	0.58 ± 0.05	8.48 ± 0.93
**j**	H, H	Me	Ph	30.51 ± 3.62	6.09 ± 0.61	30.00 ± 1.90
**k**	CH=CH-CH=CH	H	Me	6.96 ± 0.42	4.31 ± 0.36	13.50 ± 1.00
**l**	CH=CH-CH=CH	H	Et	47.00 ± 4.11	10.91 ± 2.8	34.60 ± 4.50
**m**	CH=CH-CH=CH	H	*n*-Bu	8.64 ± 0.53	6.00 ± 0.23	17.40 ± 2.50
**n**	CH=CH-CH=CH	H	*i*Pr	10.47 ± 2.04	5.52 ± 0.25	15.70 ± 0.70
**o**	CH=CH-CH=CH	H	Ph	49.96 ± 3.89	6.59 ± 0.41	71.0 ± 7.50
Carboplatin				2.9 ± 0.1	0.7 ± 0.3	3.8 ± 0.45

^1^ Compound concentration required to inhibit metabolic activity by 50%. The cells were incubated with the analogs for 48 h. Values are expressed as mean ± SEM from the concentration-response curves of at least three experiments using a nonlinear estimation (quasi-Newton algorithm) method.

## Supplementary Materials

The following are available online at https://www.mdpi.com/1420-3049/24/10/1868/s1, General procedures and characterization data for diethyl (2-(2-hydroxyarylo)-2-oxoethyl)phosphonates (**10a**–**c**), diethoxyphosphorylchromen-4-ones (**12a**–**c**) and 3-diethoxyphosphoryl-2-substituted chroman-4-ones (**13a**–**o**). Copies of ^1^H, ^13^C and ^31^P NMR spectra of all obtained compounds.
