# Supplementary material for: Synthesis and Cytotoxic Evaluation of 3-Methylidenechroman-4-ones
Source: Molecules. 2019 May 15;24(10):1868. doi: 10.3390/molecules24101868 (PMC6572547; doi:10.3390/molecules24101868)
Supplement: Supplementary file 1 [file molecules-24-01868-s001.zip › Supporting Information.docx]

Synthesis and cytotoxic evaluation of
3-methylidenechroman-4-ones

Jacek Kędzia^1^, Tomasz Bartosik^1^, Joanna Drogosz^2^, Anna Janecka^2,^, Urszula Krajewska^3^ and Tomasz Janecki^1,^*

^1^ Institute of Organic Chemistry, Lodz University of Technology, Żeromskiego 116, 90-924 Łódź, Poland; [jacek.kedzia@p.lodz.pl](mailto:jacek.kedzia@p.lodz.pl) (J.K)

^2^ Department of Biomolecular Chemistry, Medical University of Łódź, Mazowiecka 6/8, 92-215 Łódź, Poland; [anna.janecka@umed.lodz.pl](mailto:anna.janecka@umed.lodz.pl) (A.J.); [joanna.drogosz@studumed.lodz.pl](mailto:joanna.drogosz@studumed.lodz.pl) (A.D.)

^3^ Department of Pharmaceutical Biochemistry and Molecular Diagnostics, Faculty of Pharmacy, Medical University of Łódź, Muszyńskiego 1, 90-151, Łódź, Poland.

***** Correspondence: [tomasz.janecki@p.lodz.pl](mailto:tomasz.janecki@p.lodz.pl) (T.J); Tel.: +48 426313220

**Supporting Information**

Table of Contents

1. General information. 2

2. General procedures and characterization data. 2

2.1. Synthesis of diethyl (2-(2-hydroxyarylo)-2-oxoethyl)phosphonates **10a-c**. 2

2.2. Synthesis of diethyl (4-oxo-4*H*-chromen-3-yl)phosphonates **12a-c**. 3

2.3. Synthesis of diethyl (2-substituted-4-oxochroman-3-yl)phosphonates **13a-o**. 8

3. Copies of ^31^P, ^1^H and ^13^C NMR Spectra. 10

1. General information.

NMR spectra were recorded on a Bruker DPX 250 or Bruker Avance II instrument at 250.13 MHz or 700 MHz for 1H, 62.9 MHz or 176 MHz for 13C, and 101.3 MHz or 283 MHz for 31P NMR using tetramethylsilane as internal and 85% H3PO4 as external standard. 31P NMR spectra were recorded using broadband proton decoupling. IR spectra were recorded on a Bruker Alpha ATR spectrophotometer. Melting points were determined in open capillaries and are uncorrected. Column chromatography was performed on Aldrich® silica gel 60 (230–400 mesh). Thin-layer chromatography was performed with precoated TLC sheets of silica gel 60 F254 (Aldrich®). The purity of tested compounds was determined by combustion elemental analyses (CHN, elemental analyzer EuroVector 3018, Elementar Analysen systeme GmbH). MS spectra were performed on combined Waters 2695-Waters ZQ 2000 LC/MS apparatus. Reagents and starting materials were purchased from commercial vendors and used without further purification. All organic solvents were dried over appropriate drying agents and distilled prior to use. Standard syringe techniques were used for transferring dry solvents. All flasks are flame drying and flushed with argon.

2. General procedures and characterization data.

2.1. Synthesis of diethyl (2-(2-hydroxyarylo)-2-oxoethyl)phosphonates **10a-c**.

The solution of diethyl methylphosphonate **9** (1.52 g, 10.0 mmol) and ethyl salicylate **8a-c** (10.0mmol) in THF (20 mL) was cooled to -30 ^O^C and LDA generated from n-butyllithium (14 mL of 2.5 M solution in hexanes, 35.0 mmol) and diisopropylamine (4.95 mL 35.0 mmol) in THF (20.0 mL) was added dropwise maintaining the temperature bellow -10 ^O^C. The mixture was stirred for 2 hour at 0 °C and the solution was quenched by adding saturated ammonium chloride (100 mL). The water layer was extracted with DCM (3 × 50 mL), the organic layers were combined, washed with water, brine and dried over MgSO4. Filtration and concentration of the filtrate gave the crude product, which was purified by column chromatography on silica gel (ethyl DCM/Acetone = 10 : 1) to provide the desired product.

Diethyl (2-(2-hydroxyphenyl)-2-oxoethyl)phosphonate (**10a**) (2.17 g, 80%). Yellow oil. ^31^P NMR (101 MHz, Chloroform-*d*) δ 19.69.^1^H NMR (250 MHz, Chloroform-*d*) δ 1.29 (t, *J* = 7.1 Hz, 6H), 3.63 (d, *J* = 22.8 Hz, 2H), 3.94 – 4.34 (m, 4H), 6.74 – 7.07 (m, 2H), 7.49 (ddd, *J* = 8.5, 7.1, 2.0 Hz, 1H), 7.68 – 8.07 (m, 1H), 11.81 – 12.14 (m, 1H). ^13^C NMR (63 MHz, Chloroform-*d*) δ 16.29 (d, *J* = 6.3 Hz), 38.66 (d, *J* = 130.0 Hz), 62.88 (d, *J* = 6.6 Hz), 118.50 , 119.11 , 119.49 , 131.62 , 137.19 , 162.94 , 197.93 (d, *J* = 6.2 Hz). Anal. Calcd for C_12_H_17_O_5_P: C, 52.94; H, 6.29. Found: C, 52.72; H, 6.43.

Diethyl (2-(2-hydroxy-3-methylphenyl)-2-oxoethyl)phosphonate (**10b**) (2.14 g, 75%). Yellow oil. ^31^P NMR (101 MHz, Chloroform-*d*) δ 19.98 . ^1^H NMR (250 MHz, Chloroform-*d*) δ 1.20 – 1.34 (m, 6H), 2.23 (s, 3H), 3.60 (d, *J* = 22.8 Hz, 2H), 4.04 – 4.23 (m, 4H), 6.80 (t, *J* = 7.7 Hz, 1H), 7.26 (d, *J* = 0.5 Hz, 0H), 7.34 (dd, *J* = 7.3, 1.5 Hz, 1H), 7.64 (dd, *J* = 8.2, 1.6 Hz, 1H), 12.31 (s, 1H). ^13^C NMR (176 MHz, Chloroform-*d*) δ 198.10 (d, *J* = 6.3 Hz), 161.46 , 137.91 , 129.19 , 127.55 , 118.79 , 118.42 , 62.85 (d, *J* = 6.7 Hz), 38.66 (d, *J* = 130.3 Hz), 16.30 (d, *J* = 6.3 Hz), 15.50 . Anal. Calcd for C_13_H_19_O_5_P: C, 54.55; H, 6.69;. Found: C, 54.25; H, 6.52.

Diethyl (2-(3-hydroxynaphthalen-2-yl)-2-oxoethyl)phosphonate (**10c**) (2.68 g, 80%). Yellow oil. ^31^P NMR (101 MHz, Chloroform-*d*) δ 19.53 .  ^1^H NMR (250 MHz, Chloroform-*d*) δ 1.29 (t, *J* = 7.0 Hz, 6H), 3.79 (d, *J* = 22.9 Hz, 2H), 4.16 (dqd, *J* = 8.0, 7.1, 1.2 Hz, 4H), 7.33 (ddd, *J* = 8.1, 6.8, 1.2 Hz, 1H), 7.53 (ddd, *J* = 8.3, 6.7, 1.3 Hz, 1H), 7.64 – 7.70 (m, 1H), 7.80 – 7.87 (m, 1H), 8.45 – 8.49 (m, 1H), 11.26 (d, *J* = 0.5 Hz, 1H). ^13^C NMR (63 MHz, Chloroform-*d*) δ 16.31 (d, *J* = 6.4 Hz), 39.10 (d, *J* = 130.0 Hz), 62.97 (d, *J* = 6.6 Hz), 112.36 , 120.99 , 124.24 , 126.23 , 126.86 , 129.70 , 130.15 , 135.00 , 138.49 , 157.06 , 198.18 (d, *J* = 6.3 Hz). Anal. Calcd for C_16_H_19_O_5_P: C, 59.63; H, 5.94. Found: C, 59.33; H, 6.01.

2.2. Synthesis of diethyl (4-oxo-4*H*-chromen-3-yl)phosphonates **12a-c**.

The solution of diethyl (2-(2-hydroxyaryl)-2-oxoethyl)phosphonate **10a-c** (7.50 mmol) and DMFDMA (1.50mL, 1.34 g, 11.25 mmol) in dry toluene (75 mL) was heated at 80 ^O^C for 2 h. The crude mixture was diluted with toluene (20 mL) and the solvent removed. The residue was purified by column chromatography on silica gel (DCM/Acetone, 100:1), to give pure products **12a-c**.

Diethyl (4-oxo-4*H*-chromen-3-yl)phosphonate (**12a**) (1.95g, 92%). Yellow oil. C. ^31^P NMR (101 MHz, Chloroform-*d*) δ 12.57 . ^1^H NMR (700 MHz, Chloroform-*d*) δ 1.28 (t, *J* = 7.2 Hz, 6H), 4.08 – 4.28 (m, 4H), 7.37 (dd, *J* = 8.7, 6.4 Hz, 1H), 7.41 (dd, *J* = 8.5, 2.1 Hz, 1H), 7.63 (ddd, *J* = 8.7, 5.3, 1.8 Hz, 1H), 8.12 (dt, *J* = 8.0 Hz, 1H), 8.42 (d, *J* = 9.5 Hz, 1H). ^13^C NMR (176 MHz, Chloroform-*d*) δ 16.31 (d, *J* = 6.5 Hz), 62.93 (d, *J* = 5.8 Hz), 113.95 (d, *J* = 188.8 Hz), 118.22 , 124.30 (d, *J* = 8.3 Hz), 126.02 , 126.16 , 134.44 , 156.06 , 163.22 (d, *J* = 19.9 Hz), 175.17 (d, *J* = 2.5 Hz). Anal. Calcd for C_13_H_15_O_5_P: C, 55.32; H, 5.36;. Found: C, 55.63; H, 5.55.

Diethyl (8-methyl-4-oxo-4*H*-chromen-3-yl)phosphonate (**12b**) (1.98 g, 89%). Yellow oil. °C. ^31^P NMR (284 MHz, Chloroform-*d*) δ 12.39 . ^1^H NMR (700 MHz, Chloroform-*d*) δ 1.33 (t, *J* = 7.0 Hz, 7H), 2.45 (s, 3H), 4.14 – 4.29 (m, 4H), 7.30 (t, *J* = 7.6 Hz, 1H), 7.51 (dd, *J* = 7.3, 1.7 Hz, 1H), 8.01 (dd, *J* = 7.9, 1.7 Hz, 1H), 8.51 (d, *J* = 9.5 Hz, 1H). ^13^C NMR (176 MHz, Chloroform-*d*) δ 15.51 , 16.43 (d, *J* = 6.4 Hz), 63.03 (d, *J* = 5.7 Hz), 113.74 (d, *J* = 189.0 Hz), 123.71 , 124.38 (d, *J* = 8.3 Hz), 125.75 , 127.89 , 135.51 , 154.71 , 163.11 (d, *J* = 20.0 Hz), 175.70 (d, *J* = 2.2 Hz). Anal. Calcd for C_14_H_17_O_5_P: C, 56.76; H, 5.78;. Found: C, 56.45; H, 5.89.

Diethyl (4-oxo-4*H*-benzo[g]chromen-3-yl)phosphonate (**12c**) (1.94g, 78%). White crystal, mp 158-160 °C. ^31^P NMR (101 MHz, Chloroform-*d*) δ 13.01 . ^1^H NMR (250 MHz, Chloroform-*d*) δ 1.38 (t, *J* = 7.1 Hz, 6H), 4.07 – 4.49 (m, 4H), 7.56 (ddd, *J* = 8.1, 6.8, 1.3 Hz, 1H), 7.65 (ddd, *J* = 8.5, 6.8, 1.4 Hz, 1H), 7.89 – 7.96 (m, 2H), 8.05 (dd, *J* = 8.3, 1.4 Hz, 1H), 8.57 (d, *J* = 9.6 Hz, 1H), 8.76 – 8.83 (m, 1H). ^13^C NMR (176 MHz, Chloroform-*d*) δ 16.41 (d, *J* = 6.5 Hz), 62.98 (d, *J* = 5.8 Hz), 112.18 (d, *J* = 189.9 Hz), 114.82 , 122.66 (d, *J* = 8.3 Hz), 126.62 , 127.37 , 127.53 , 129.30 , 129.69 (d, *J* = 3.7 Hz), 130.68 , 135.91 , 152.11 , 164.32 (d, *J* = 22.4 Hz), 176.06 . Anal. Calcd for C_17_H_17_O_5_P: C, 61.45; H, 5.16;. Found: C, 61.22; H, 5.32.

2.3. Synthesis of diethyl (2-substituted-4-oxochroman-3-yl)phosphonates **13a-o**.

To the solution of diethoxyphosphorylchromen-4-one **12a-c** (0.5 mmol) in dry THF (7.5 mL) provided with ice-water bath, Cu_2_I_2_ (58.6 mg, 0.15mml) was added in one portion. After 15 minutes Grignard reagent (1.5 mmol solution in THF) was added dropwise in argon atmosphere. The mixture was stirred at room temperature overnight. Next, saturated solutions of NH_4_Cl (10 mL) and DCM (10 mL) were added. After separation, the water layer was washed with DCM (2x10 mL). Combined organic extracts were washed with brine (10 mL) and dried over MgSO_4_. The solvents were evaporated under reduced pressure and the resulting crude product was purified by column chromatography (eluent DCM : Acetone 20:1).

Diethyl (2-methyl-4-oxochroman-3-yl)phosphonate (**13a**) (117.3 mg, 84%). Yellow oil. ^31^P NMR (284 MHz, Chloroform-*d*) δ 17.77 (*cis*), 19.09 (*trans*), 23.10 (enol). ^1^H NMR (700 MHz, Chloroform-*d*) δ 1.01 (t, *J* = 7.1 Hz, 3H, trans), 1.06 (t, *J* = 7.0 Hz, 3H, cis), 1.27 (t, *J* = 7.1 Hz, 3H, trans), 1.41 (dd, *J* = 6.8, 1.6 Hz, 6H, trans), 1.70 (d, *J* = 6.7 Hz, 3H, cis), 3.03 (dd, *J* = 25.2, 1.8 Hz, 1H, trans), 3.23 (dd, *J* = 24.5, 3.5 Hz, 1H, cis), 3.90 – 4.16 (m, 6H, trans+cis), 4.79 (dddd, *J* = 33.4, 6.7, 3.5 Hz, 1H, cis), 5.26 (dqd, *J* = 12.0, 6.8, 1.9 Hz, 1H, trans), 6.90 (dd, *J* = 8.3, 1.0 Hz, 1H, trans), 6.92 – 6.99 (m, 2H, trans), 7.44 (m, 3H, cis), 7.84 (m, 2H, trans+cis).^13^C NMR (176 MHz, Chloroform-*d*) δ 16.03 (d, *J* = 6.4 Hz, trans), 16.10 (d, *J* = 6.4 Hz, cis), 16.27 (d, *J* = 6.2 Hz, trans), 16.33 (d, *J* = 6.1, 1H, cis), 18.35 (d, *J* = 1.9 Hz, cis), 19.39 (d, *J* = 16.5 Hz, trans), 51.91 (d, *J* = 129.7 Hz, cis), 52.85 (d, *J* = 126.6 Hz, trans), 62.51 (d, *J* = 6.4 Hz, cis), 62.74 (d, *J* = 6.5 Hz, cis), 62.81 (d, *J* = 6.9 Hz, trans), 63.19 (d, *J* = 6.9 Hz, trans), 73.49 (d, *J* = 5.4 Hz, trans), 74.98 (d, *J* = 5.1 Hz, cis), 117.78 (cis), 118.36 (trans), 120.59 (trans), 120.67 (cis), 121.24 (trans), 121.41 (cis), 126.95 (trans), 127.48 (cis), 136.23 (cis), 136.61 (trans) , 158.80 (trans), 161.14 (cis), 186.50 (d, *J* = 4.8 Hz, trans), 187.85 (d, *J* = 4.2 Hz, cis).ESI-MS [M+H]^+^ = 299.6. Anal. Calcd for C_14_H_19_O_5_P: C, 56.38; H, 6.42;. Found: C, 56.12; H, 6.23.

Due to small amount of the enol form, the signals of enol form in the ^1^H and ^13^C NMR spectra have not been assigned.

Diethyl (2-ethyl-4-oxochroman-3-yl)phosphonate (**13b**) (106.2 mg, 68%). Yellow oil. ^31^P NMR (101 MHz, Chloroform-*d*) δ 18.07 (cis), 19.78 (trans), 23.49 (enol).^1^H NMR (250 MHz, Chloroform-*d*) δ 0.91 – 1.09 (m, 6H, trans+cis), 1.28 (t, *J* = 7.1, Hz, 6H, trans+cis), 1.52 (m, 1H, trans), 1.78 – 2.14 (m, 1H, trans+cis), 2.14 – 2.34 (m, 1H, cis), 3.08 (dd, *J* = 25.5, 1.3 Hz, 1H, trans), 3.27 (dd, *J* = 24.4, 3.3 Hz, 1H, cis), 3.83 – 4.19 (m, 4H, trans+cis), 4.48 (dddd, *J* = 35.5, 8.9, 5.3, 3.4 Hz, cis), 4.99 (dddd, *J* = 12.7, 9.7, 5.2, 1.3 Hz, 1H, trans), 6.86 – 7.09 (m, 2H, trans+cis), 7.39 – 7.53 (m, 1H, trans+cis), 7.84 (m, 1H, trans+cis), 11.01 (d, *J* = 1.1 Hz, 0H). ^13^C NMR (63 MHz, Chloroform-*d*) δ 10.08 (trans), 10.35 (cis), 15.95 (d, *J* = 6.2 Hz, trans), 16.21 (d, *J* = 6.3 Hz, trans), 25.32 (cis), 25.87 (d, *J* = 16.3 Hz, trans), 51.79 (d, *J* = 130.0 Hz, cis), 52.15 (d, *J* = 126.6 Hz, trans), 62.46 (d, *J* = 6.2 Hz, cis), 62.86 (d, *J* = 6.9 Hz, trans), 63.14 (d, *J* = 6.6 Hz, trans), 78.38 (d, *J* = 6.0 Hz, trans), 117.68 (cis), 118.26 (trans), 120.82 (cis), 120.89 (trans), 121.16 (trans), 121.34 (cis), 126.85 (trans), 127.44 (cis), 136.16 (cis), 136.56 (trans), 158.56 (trans), 161.21 (cis), 186.33 (d, *J* = 4.6 Hz, trans), 187.84 (d, *J* = 5.6 Hz, cis). ESI-MS [M+H]^+^ = 313.5. Anal. Calcd for C_15_H_21_O_5_P: C, 57.69; H, 6.78;. Found: C, 57.55; H, 6.51.

Due to small amount of the enol form, the signals of enol form in the ^1^H and ^13^C NMR spectra have not been assigned. Also, for the same reason, not all signals of cis form were assigned.

Diethyl (2-ethyl-4-oxochroman-3-yl)phosphonate (**13c**) (144.6 mg, 85%). Yellow oil. ^31^P NMR (284 MHz, Chloroform-*d*) δ 17.79 (cis), 19.39 (trans), 23.03 (enol). ^1^H NMR (700 MHz, Chloroform-*d*) δ 0.82 (t, *J* = 7.3 Hz, 3H, trans), 0.90 (t, *J* = 7.2 Hz, 3H, cis), 1.00 (t, *J* = 7.1, Hz, 3H, trans), 1.05 (t, *J* = 7.1 Hz, 3H, cis), 1.20 – 1.31 (m, 3H, trans+cis), 1.31 – 1.42 (m, 2H, trans+cis), 1.45 – 1.54 (m, 1H, trans+cis), 1.73 – 1.79 (m, 1H, trans), 1.87 (dtd, *J* = 14.6, 9.5, 5.3 Hz, 1H, trans), 1.99 (ddd, *J* = 14.3, 10.3, 5.2 Hz, 1H, cis), 2.23 (dtd, *J* = 14.4, 9.3, 5.0 Hz, 1H, cis), 3.07 (dd, *J* = 25.4, 1.2 Hz, 1H, trans), 3.25 (dd, *J* = 24.5, 3.3 Hz, 1H, cis), 3.89 – 4.06 (m, 4H, trans+cis), 4.10 (m, 4H, trans+cis), 4.57 (dddd, *J* = 35.4, 8.7, 4.9, 3.3 Hz, 1H, cis), 5.08 (dddd, *J* = 12.7, 9.7, 5.2, 1.3 Hz, 1H, trans), 6.92 (dd, *J* = 8.3, 1.0 Hz, 1H, trans), 6.94 – 6.99 (m, 2H, trans+cis), 7.45 (m, 1H, trans+cis), 7.84 (dd, *J* = 7.8, 1.7 Hz, 1H, trans), 7.85 (dd, *J* = 7.9, 1.8 Hz, 1H, cis). ^13^C NMR (176 MHz, Chloroform-*d*) δ 13.83 (trans), 13.92 (cis), 15.94 (d, *J* = 6.4 Hz, trans), 16.01 (d, *J* = 6.7 Hz, cis), 16.20 (d, *J* = 6.1 Hz, trans), 16.25 (d, *J* = 5.9 Hz, cis), 22.05 (trans), 22.25 (cis), 27.69 (trans), 28.01 (cis), 31.69 (cis), 32.29 (d, *J* = 15.9 Hz, trans), 51.06 (d, *J* = 129.3 Hz, cis), 51.70 (d, *J* = 126.8 Hz, trans), 62.40 (d, *J* = 6.4 Hz, cis), 62.69 (d, *J* = 6.6 Hz, cis), 62.81 (d, *J* = 6.8 Hz, trans), 63.13 (d, *J* = 6.5 Hz, trans), 77.09 (trans), 79.10 (d, *J* = 5.3 Hz, cis), 117.69 (cis), 118.26 (trans), 120.84 (cis), 120.88 (trans), 121.12 (trans), 121.32 (cis), 126.86 (trans), 127.46 (cis), 136.12 (cis), 136.54 (trans), 158.62 (trans), 161.25 (cis), 186.43 (d, *J* = 4.8 Hz, trans), 187.93 (d, *J* = 3.8 Hz, cis). ESI-MS [M+H]^+^ = 341.7. Anal. Calcd for C_17_H_25_O_5_P: , 59.99; H, 7.40;. Found: , 60.08; H, 7.21.

Due to small amount of the enol form, the signals of enol form in the ^1^H and ^13^C NMR spectra have not been assigned.

Diethyl (2-isopropyl-4-oxochroman-3-yl)phosphonate (**13d**) (107.6 mg, 66%). Yellow oil. ^31^P NMR (101 MHz, Chloroform-*d*) δ 17.64 (cis), 20.36 (trans), 23.97 (enol). ^1^H NMR (700 MHz, Chloroform-*d*) δ 0.88 (d, *J* = 6.7 Hz, 3H, trans), 0.96 – 0.97 (m, 3H, trans), 0.98 (t, *J* = 7.0, Hz, 3H, trans+cis), 1.13 (d, *J* = 6.5 Hz, 3H, cis), 1.27 (t, *J* = 7.1, Hz, 3H, trans+cis), 1.29 – 1.33 (m, 3H, cis), 2.01 (dpd, *J* = 10.0, 6.6, 1.0 Hz, 1H, trans), 2.77 (dp, *J* = 10.3, 6.6 Hz, 1H, cis), 3.27 (dd, *J* = 25.8, 1.1 Hz, 1H, trans), 3.33 (dd, *J* = 23.9, 2.8 Hz, 1H, cis), 3.84 – 3.98 (m, 4H, cis), 3.98 – 4.12 (m, 4H, trans), 4.13 – 4.24 (m, 1H, cis), 4.61 (ddd, *J* = 13.0, 10.0, 1.2 Hz, 1H, trans), 6.92 (dd, *J* = 8.3, 1.0 Hz, 1H, trans), 6.93 – 6.99 (m, 1H, trans+cis), 7.45 (ddd, *J* = 8.3, 7.2, 2.0 Hz, 1H, trans+cis), 7.81 (dd, *J* = 7.8, 1.7 Hz, 1H, trans), 7.85 (dd, *J* = 8.1, 1.7 Hz, 1H, cis). ^13^C NMR (176 MHz, Chloroform-*d*) δ 15.93 (d, *J* = 6.5 Hz, cis), 16.00 (d, *J* = 6.3 Hz, trans), 16.23 (d, *J* = 6.3 Hz, cis), 16.27 (d, *J* = 6.3 Hz, trans), 18.58 (cis), 18.60 (trans), 19.20 (trans), 19.90 (cis), 29.73 (d, *J* = 15.2 Hz, trans), 30.27 (d, *J* = 2.5 Hz, cis), 48.89 (d, *J* = 125.6 Hz, trans), 49.37 (d, *J* = 125.4 Hz, cis), 62.43 (d, *J* = 6.6 Hz, cis), 62.72 (d, *J* = 6.5 Hz, cis), 62.84 (d, *J* = 6.7 Hz, trans), 63.23 (d, *J* = 6.5 Hz, trans), 82.70 (d, *J* = 6.2 Hz, trans), 85.15 (d, *J* = 7.1 Hz, cis), 117.53 (cis), 118.12 (trans), 120.79 (cis), 121.15 (trans), 121.42 (trans), 126.84 (trans), 127.64 (cis), 136.09 (cis), 136.59 (trans), 159.02 (trans), 161.99 (cis), 186.43 (d, *J* = 4.5 Hz, trans), 188.32 (d, *J* = 3.6 Hz, cis). ESI-MS [M+H]^+^ = 327.5. Anal. Calcd for C_16_H_23_O_5_P: C, 58.89; H, 7.10;. Found: C, 59.02; H, 7.29.

Due to small amount of the enol form, the signals of enol form in the ^1^H and ^13^C NMR spectra have not been assigned.

Diethyl (4-oxo-2-phenylchroman-3-yl)phosphonate (**13e**) (115.3 mg, 64%). Yellow oil. ^31^P NMR (101 MHz, Chloroform-*d*) δ 16.28 (cis), 19.49 (trans), 22.71 (enol). ^1^H NMR (Chloroform-*d*, 700 MHz): δ (ppm) 0.83 (t, *J*=7.0 Hz, 3H, cis), 0.98 (t, *J*=7.1 Hz, 3H, enol), 1.01 (t, *J*=7.1 Hz, 3H, enol), 1.07 (t, *J*=7.1 Hz, 3H, trans), 1.30 (t, *J*=7.1 Hz, 3H, trans), 1.39 (t, *J*=7.1 Hz, 3H, cis), 3.40 – 3.47 (m, 1H, enol), 3.60 (dd, *J*=24.3, 3.5 Hz, 1H, cis), 3.72 (dd, *J*=25.7, 2.0 Hz, 1H, trans), 3.78 – 3.85 (m, 1H, cis+enol), 3.88 (m, 1H, cis), 4.03 (m, 1H, trans), 4.08 – 4.18 (m, 3H, trans), 5.77 (dd, *J*=38.4, 3.5 Hz, 1H, cis), 5.81 (d, *J*=8.3 Hz, 1H, enol), 6.23 (dd, *J*=12.6, 1.9 Hz, 1H, trans), 6.71 (d, *J*=8.2 Hz, 1H, enol), 6.95 (dd, *J*=8.1, 8.0, 1.1 Hz, 1H, trans), 7.02 (d, *J*=8.3 Hz, 1H, trans), 7.06 (ddd, *J*=8.0, , 8.0, 1.1 Hz, 1H, cis), 7.13 (d, *J*=8.3 Hz, 1H, cis), 7.18 – 7.24 (m, 1H, trans), 7.24 – 7.28 (m, 3H, trans), 7.28 – 7.31 (m, 2H, trans), 7.31 – 7.35 (m, 1H, cis), 7.37 – 7.43 (m, 3H, enol), 7.45 – 7.49 (m, 1H, trans), 7.51 – 7.54 (m, 1H, enol), 7.55 – 7.58 (m, 2H, cis), 7.67 (dd, *J*=7.8, 1.6 Hz, 1H, enol), 7.80 (dd, *J*=7.9, 1.7 Hz, 1H, trans), 7.95 (dd, *J*=7.9, 1.7 Hz, 1H, cis), 11.30 (s, 1H, enol). ^13^C NMR (Chloroform-*d*, 176 MHz): δ (ppm) 16.02 (d, *J*=6.4 Hz, ), 16.22 (d, *J*=6.3 Hz, H), 51.12 (d, *J*=126.2 Hz, H), 63.17 (dd, *J*=79.5, 6.8 Hz, H), 77.64 (d, *J*=4.5 Hz, H), 118.12 , H, 121.47 , H, 125.82 , H, 126.45 , H, 126.97 , H, 128.30 (d, *J*=3.2 Hz, H), 128.38 , H, 128.73 , H, 136.70 , H, 137.75 (d, *J*=16.2 Hz, H), 159.33 , H, 185.95 (d, *J*=4.5 Hz, H).. ESI-MS [M+H]^+^ = 361.6. Anal. Calcd for C_19_H_21_O_5_P: C, 63.33; H, 5.87;. Found: C, 63.52; H, 5.99.

Due to small amount of the enol form, the signals of enol form in the ^1^H and ^13^C NMR spectra have not been assigned.

Diethyl (2,8-dimethyl-4-oxochroman-3-yl)phosphonate (**13f**) (115.5 mg, 74%). Yellow oil. ^31^P NMR (Chloroform-*d*, 101 MHz): δ (ppm) 18.72 (cis) , 20.08 (trans), 23.98 (enol). ^1^H NMR (Chloroform-*d*, 700 MHz): δ (ppm) 1.03 (t, *J*=7.0, Hz, 3H, trans), 1.10 (t, *J*=7.1 Hz, 3H, cis), 1.32 (m, Hz, 3H, trans+cis), 1.45 (dd, *J*=6.7, 1.6 Hz, 3H, trans), 1.76 (dd, *J*=6.7, 0.7 Hz, 1H, cis), 2.22 (s, 3H, trans), 2.24 (s, Hz, 3H, cis), 3.06 (dd, *J*=25.1, 1.9 Hz, 1H, trans), 3.27 (dd, *J*=24.4, 3.6 Hz, 1H, cis), 3.87 – 4.20 (m, 4H, trans+cis), 4.84 (dddd, *J*=32.9, 9.9, 6.6, 3.6 Hz, 1H, cis), 5.37 (dddd, *J*=12.1, 8.6, 6.8, 1.9 Hz, 1H, trans), 6.90 (m, Hz, 1H, trans+cis, 7.35 (m, 1H, trans+cis), 7.74 (m, 1H, trans+cis). ^13^C NMR (Chloroform-*d*, 176 MHz): δ (ppm) 15.36 (cis), 15.45 (trans), 15.77 (d, *J*=6.4 Hz, trans), 15.86 (d, *J*=6.4 Hz, cis), 16.06 (d, *J*=6.1 Hz, trans), 16.11 (d, *J*=5.5 Hz, cis), 18.14 (cis), 19.34 (d, *J*=16.4 Hz, trans), 51.51 (d, *J*=130.6 Hz, cis), 52.45 (dd, *J*=126.5 Hz, trans), 62.22 (d, *J*=6.4 Hz, cis), 62.38 (d, *J*=6.5 Hz, cis), 62.55 (d, *J*=6.6 Hz, trans), 62.84 (d, *J*=6.5 Hz, trans), 73.11 (d, *J*=5.2 Hz, trans), 74.56 (d, *J*=5.0 Hz, cis), 120.03 (trans), 120.09 (cis), 120.40 (trans), 120.56 (cis), 124.32 (trans), 124.83 (cis), 126.73 (cis), 127.27 (trans), 136.80 (trans), 137.18 (cis), 156.70 (trans), 159.12 (cis), 186.58 (d, *J*=4.6 Hz, trans), 187.90 (d, *J*=4.3 Hz, cis). ESI-MS [M+H]^+^ = 313.2. Anal. Calcd for C_15_H_21_O_5_P: C, 57.69; H, 6.78;. Found: C, 57.81; H, 6.53.

Due to small amount of the enol form, the signals of enol form in the ^1^H and ^13^C NMR spectra have not been assigned.

Diethyl (2-ethyl-8-methyl-4-oxochroman-3-yl)phosphonate (**13g**) (124.0 mg, 76%). Yellow oil. ^31^P NMR (Chloroform-*d*, 101 MHz): δ (ppm) 18.65 (cis), 20.32 (trans), 23.99 (enol). ^1^H NMR (Chloroform-*d*, 700 MHz): δ (ppm) 0.99 (t, *J*=7.3 Hz, 3H, trans), 1.01 (t, *J*=7.1 Hz, 3H, trans), 1.06 – 1.09 (m, 3H, cis), 1.10 (t, *J*=7.4 Hz, 3H, cis), 1.29 – 1.34 (m, 3H, trans+cis), 1.47 – 1.60 (m, 1H, trans), 1.91 (ddq, *J*=14.4, 10.1, 7.2 Hz, 1H, trans), 2.00 – 2.07 (m, 1H, cis), 2.24 (d, *J*=1.4 Hz, 3H, trans), 2.26 (d, *J*=0.7 Hz, 3H, cis), 2.30 – 2.38 (m, 1H, cis), 3.10 (dd, *J*=25.4, 1.3 Hz, 1H, trans), 3.30 (dd, *J*=24.4, 3.4 Hz, 1H, cis), 3.91 – 4.09 (m, 2H, trans+cis), 4.10 – 4.18 (m, 2H, trans+cis), 4.53 (dddd, *J*=34.9, 9.3, 4.5, 1.1 Hz, 1H, cis), 5.08 (dddd, *J*=12.8, 10.1, 4.8, 1.3 Hz, 1H, cis), 6.89 (dd, *J*=7.6, 7.6 Hz, 1H,, trans), 6.91 (dd, *J*=7.7, 7.7 Hz, 1H, cis), 7.34 (dddd, *J*=6.2, 5.4, 1.7, 0.9 Hz, 1H), 7.72 (ddd, *J*=7.9, 1.8, 0.7 Hz, 1H, trans), 7.74 (ddd, *J*=7.9, 1.7, 0.7 Hz, 1H, trans). ^13^C NMR (Chloroform-*d*, 176 MHz): δ (ppm) 10.23 (trans), 10.47 (cis), 15.36 (cis), 15.43 (trans), 15.82 (d, *J*=6.4 Hz, trans), 15.93 (d, *J*=6.7 Hz, cis), 16.15 (d, *J*=6.3 Hz, trans), 16.19 (d, *J*=6.4 Hz, cis), 25.24 (cis), 25.83 (d, *J*=16.5 Hz, trans), 50.82 (d, *J*=129.4 Hz, cis), 51.51 (d, *J*=125.8 Hz, trans), 62.28 (d, *J*=6.4 Hz, cis), 62.47 (d, *J*=6.2 Hz, cis), 62.67 (d, *J*=6.1 Hz, trans), 62.94 (d, *J*=6.7 Hz, trans), 78.27 (d, *J*=5.7 Hz, trans), 80.24 (d, *J*=5.2 Hz, cis), 120.42 (cis), 120.50 (trans), 120.51 (trans), 120.67 (cis), 124.43 (trans), 124.98 (cis), 126.85 (cis), 127.27 (trans), 136.84 (cis), 137.27 (trans), 156.54 (trans), 159.24 (cis), 186.63 (d, *J*=4.9 Hz, trans), 188.08 (d, *J*=4.2 Hz, cis). ESI-MS [M+H]^+^ = 327.2. Anal. Calcd for C_16_H_23_O_5_P: C, 58.89; H, 7.10;. Found: C, 59.07; H, 7.27.

Due to small amount of the enol form, the signals of enol form in the ^1^H and ^13^C NMR spectra have not been assigned.

Diethyl (2-butyl-8-methyl-4-oxochroman-3-yl)phosphonate (**13h**) (147.1 mg, 83%). Yellow oil. ^31^P NMR (Chloroform-*d*, 101 MHz): δ (ppm) 18.41 (cis), 20.04 (trans), 23.61 (enol). ^1^H NMR (Chloroform-*d*, 700 MHz): δ (ppm) 0.85 (t, *J*=7.2 Hz, 3H, trans), 0.93 (t, *J*=7.2 Hz, 3H, cis), 0.98 – 1.02 (m, 3H, trans), 1.07 (t, *J*=7.0 Hz, 3H, cis), 1.26 (m, 2H, cis), 1.32 (t, *J*=7.1 Hz, 3H, trans+cis), 1.35 – 1.42 (m, 2H, trans+cis), 1.46 – 1.52 (m, 1H, trans), 1.53 – 1.59 (m, 1H, cis), 1.84 – 1.92 (m, 1H, trans), 1.94 – 2.02 (m, 1H, cis), 2.22 (s, 3H, trans), 2.25 (s, 3H, cis), 2.29 – 2.35 (m, 1H, cis), 3.08 (dd, *J*=25.2, 1.3 Hz, 1H, trans), 3.27 (dd, *J*=24.4, 3.4 Hz, 1H, cis), 3.90 – 4.07 (m, 2H, trans+cis), 4.09 – 4.17 (m, 2H, trans+cis), 4.60 (dddd, *J*=34.7, 9.8, 7.6, 3.9 Hz, 1H, cis), 5.16 (dddd, *J*=12.8, 10.0, 4.9, 1.3 Hz, 1H, trans), 6.89 (dd, *J*=7.5, 7.5 Hz, 1H, trans), 6.91 (dd, *J*=7.7, 7.7 Hz, 1H, cis), 7.34 (m, 1H, trans+cis), 7.72 (dd, *J*=7.8, 1.7 Hz, 1H, trans), 7.73 (dd, *J*=8.3, 1.9 Hz, 1H, cis). ^13^C NMR (Chloroform-*d*, 176 MHz): δ (ppm) 13.63 (trans), 13.74 (cis), 15.23 (cis), 15.29 (trans), 15.66 (d, *J*=6.3 Hz, trans), 15.76 (d, *J*=6.4 Hz, cis), 15.98 (d, *J*=6.3 Hz, trans), 16.02 (d, *J*=5.9 Hz, cis), 21.77 (trans), 22.00 (cis), 27.66 (trans), 27.98 (cis), 31.39 (cis), 32.04 (d, *J*=16.0 Hz, trans), 50.82 (d, *J*=131.0 Hz, cis), 51.44 (d, *J*=124.5 Hz, trans), 62.10 (d, *J*=6.1 Hz, cis), 62.31 (d, *J*=6.2 Hz, cis), 62.51 (d, *J*=6.8 Hz, trans), 62.77 (d, *J*=7.2 Hz, trans), 76.68 (d, *J*=5.0 Hz, trans), 78.68 (d, *J*=5.2 Hz, cis), 120.26 (cis), 120.32 (trans), 120.51 (cis), 124.25 (trans), 124.81 (cis), 126.66 (cis), 127.08 (trans), 136.66 (cis), 137.11 (trans), 156.42 (trans), 159.14 (cis), 186.46 (d, *J*=4.8 Hz, trans), 187.89 (d, *J*=4.0 Hz, cis). ESI-MS [M+H]^+^ = 355.3. Anal. Calcd for C_18_H_27_O_5_P: C, 61.01; H, 7.68;. Found: C, 60.88; H, 7.52.

Due to small amount of the enol form, the signals of enol form in the ^1^H and ^13^C NMR spectra have not been assigned.

Diethyl (2-isopropyl-8-methyl-4-oxochroman-3-yl)phosphonate (**13i**) (119.1 mg, 70%). White crystal, mp 60-63 °C. ^31^P NMR (Chloroform-*d*, 101 MHz): δ (ppm) 18.10 (cis), 20.86 (trans), 24.47 (enol). ^1^H NMR (Chloroform-*d*, 700 MHz): δ (ppm) 0.81 (d, *J*=6.7 Hz, 3H, trans), 0.84 – 0.90 (m, 6H, trans+cis), 0.92 (d, *J*=6.7 Hz, 3H, cis), 1.07 (d, *J*=6.5 Hz, 3H, cis), 1.18 (m, 3H, trans+cis), 1.85 – 1.95 (m, 1H, trans), 2.13 (s, 3H, trans), 2.15 (s, 3H, cis), 2.74 (dp, *J*=10.3, 6.6 Hz, 1H, cis), 3.19 (dd, *J*=26.0, 1.2 Hz, 1H, trans), 3.22 (dd, *J*=23.8, 2.8 Hz, 1H, cis), 3.75 – 4.04 (m, 6H), 4.58 (ddd, *J*=12.9, 10.0, 1.2 Hz, 1H, trans), 6.76 (dd, *J*=7.6, 7.6 Hz, 1H, trans), 6.78 (dd, *J*=7.6, 7.6 Hz, 1H, cis), 7.22 (m, 1H, trans+cis), 7.58 (dd, *J*=7.9, 1.7 Hz, 1H, trans), 7.61 (dd, *J*=7.9, 1.7 Hz, 1H, cis). ^13^C NMR (Chloroform-*d*, 176 MHz): δ (ppm) 15.32 (trans), 15.68 (cis), 15.72 (d, *J*=6.4 Hz, trans+cis), 16.02 (d, *J*=6.2 Hz, cis), 16.04 (d, *J*=6.4 Hz, trans), 18.32 (cis), 18.44 (trans), 19.17 (trans), 19.77 (cis), 29.30 (d, *J*=15.3 Hz, trans), 30.17 (cis), 48.91 (d, *J*=125.8 Hz, trans), 49.02 (d, *J*=125.4 Hz, cis), 62.19 (d, *J*=6.3 Hz, cis), 62.35 (d, *J*=6.6 Hz, cis), 62.57 (d, *J*=6.8 Hz, trans), 62.90 (d, *J*=6.6 Hz, trans), 82.43 (d, *J*=6.2 Hz, trans), 84.77 (d, *J*=7.2 Hz, cis), 120.16 (cis), 120.38 (trans), 120.66 (cis), 120.82 (trans), 124.26 (trans), 125.03 (cis), 126.62 (cis), 126.87 (trans), 136.63 (cis), 137.20 (trans), 156.81 (trans), 159.88 (cis), 186.48 (d, *J*=4.6 Hz, trans), 188.29 (d, *J*=3.6 Hz, cis). ESI-MS [M+H]^+^ = 341.7. Anal. Calcd for C_17_H_25_O_5_P: C, 59.99; H, 7.40;. Found: C, 60.07; H, 7.23.

Due to small amount of the enol form, the signals of enol form in the ^1^H and ^13^C NMR spectra have not been assigned.

Diethyl (8-methyl-4-oxo-2-phenylchroman-3-yl)phosphonate (**13j**) (157.2 mg, 84%). White crystal, mp 54-56 °C.^31^P NMR (Chloroform-*d*, 101 MHz): δ (ppm) 16.73 (cis), 20.05 (trans), 23.23 (cis). ^1^H NMR (Chloroform-*d*, 700 MHz): δ (ppm) 1.00 (m, 6H, cis), 1.04 (t, *J*=7.1 Hz, 3H, trans), 1.32 (t, *J*=7.1 Hz, 3H, trans), 2.30 (s, 3H, trans), 2.36 (s, 3H, cis), 3.59 (dd, *J*=24.2, 3.5 Hz, 1H, cis), 3.72 (dd, *J*=25.7, 1.8 Hz, 1H, trans), 3.79 – 3.90 (m, 1H, cis), 4.00 (m, 1H, trans+cis), 4.06 – 4.19 (m, 3H, trans+cis), 5.77 (dd, *J*=39.1, 3.4 Hz, 1H, cis), 6.30 (dd, *J*=12.8, 1.8 Hz, 1H, trans), 6.86 (dd, *J*=7.6, 7.6 Hz, 1H, trans), 7.19 – 7.24 (m, 1H, cis), 7.26 (m, 1H, trans+cis), 7.33 (d, *J*=7.2 Hz, 1H, cis), 7.66 (dd, *J*=7.9, 1.7 Hz, 1H, trans). ^13^C NMR (Chloroform-*d*, 176 MHz): δ (ppm) 15.57 (cis), 15.65 (trans), 15.84 (d, *J*=6.3 Hz, trans+cis), 16.11 (d, *J*=6.3 Hz, trans+cis), 50.80 (d, *J*=126.2 Hz, trans), 52.87 (d, *J*=127.5 Hz, cis), 62.02 (d, *J*=7.0 Hz, cis), 62.39 (d, *J*=6.2 Hz, cis), 62.79 (d, *J*=6.1 Hz, trans), 63.14 (d, *J*=6.1 Hz, trans), 77.34 (trans), 78.57 (d, *J*=7.4 Hz, cis), 120.79 (trans), 121.07 (trans), 124.46 (trans), 125.57 (cis), 126.05 9trans), 126.92 (trans), 127.52 (trans), 128.07 (trans), 128.19 (trans), 128.60 (trans), 137.37 (trans), 137.80 (d, *J*=16.5 Hz, trans), 157.19 (trans), 159.44 (cis), 186.08 (d, *J*=4.6 Hz, trans)), 187.83 (cis).ESI-MS [M+H]^+^ = 357.7. Anal. Calcd for C_20_H_23_O_5_P: C, 64.17; H, 6.19;. Found: C, 64.31; H, 6.32.

Due to small amount of the enol form, the signals of enol form in the ^1^H and ^13^C NMR spectra have not been assigned. Also, for the same reason, not all signals of cis form were assigned.

Diethyl (2-methyl-4-oxo-3,4-dihydro-2*H*-benzo[*g*]chromen-3-yl)phosphonate (**13k**) (140.1 mg, 81%). White crystal, mp 78-81 °C. ^31^P NMR (Chloroform-*d*, 284 MHz): δ (ppm) 17.87 (cis), 19.18 (trans), 22.79 (enol). ^1^H NMR (Chloroform-*d*, 700 MHz): δ (ppm) 1.01 (t, *J*=7.1 Hz, 3H, trans), 1.33 (t, *J*=7.1 Hz, 3H, trans), 1.46 (dd, *J*=6.8, 1.7 Hz, 3H, trans), 3.18 (dd, *J*=25.2, 1.7 Hz, 1H, trans), 3.98 (m, 1H, trans), 4.04 – 4.20 (m, 3H, trans), 5.35 (dqd, *J*=11.7, 6.8, 1.7 Hz, 1H, trans), 7.31 (s, 1H, trans), 7.35 (ddd, *J*=8.1, 6.7, 1.2 Hz, 1H, trans), 7.50 (ddd, *J*=8.2, 6.7, 1.2 Hz, 1H, trans), 7.69 (d, *J*=8.5 Hz, 1H, trans), 7.87 (d, *J*=8.4 Hz, 1H, trans), 8.50 (s, 1H, trans). ^13^C NMR (Chloroform-*d*, 176 MHz): δ (ppm) 16.18 (d, *J*=6.2 Hz, trans), 16.37 (d, *J*=5.9 Hz, trans), 19.86 (d, *J*=16.0 Hz, trans), 53.36 (d, *J*=129.9 Hz, trans), 62.95 (d, *J*=6.6 Hz, trans), 63.39 (d, *J*=6.6 Hz, trans), 73.32 (d, *J*=5.4 Hz, trans), 113.43 (trans), 121.54 (trans), 124.85 (trans), 126.66 (trans), 128.47 (trans), 129.27 (trans), 129.40 (trans), 130.19 (trans), 138.37 (trans), 153.95 (trans), 187.41 (d, *J*=5.1 Hz, trans). ESI-MS [M+H]^+^ = 349.1. Anal. Calcd for C_18_H_21_O_5_P: C, 62.07; H, 6.08;. Found: C, 61.89; H, 5.97.

Due to small amount of the enol form, the signals of enol form in the ^1^H and ^13^C NMR spectra have not been assigned.

Diethyl (2-ethyl-4-oxo-3,4-dihydro-2*H*-benzo[*g*]chromen-3-yl)phosphonate (**13l**) (115.9 mg, 64%). White crystal, mp 84-88 °C.^31^P NMR (Chloroform-*d*, 101 MHz): δ (ppm) 17.84 (cis), 19.48 (trans), 22.82 (enol). ^1^H NMR (Chloroform-*d*, 700 MHz): δ (ppm) 0.98 – 1.04 (m, 6H), 1.34 (t, *J*=7.1 Hz, 3H), 1.59 (dqd, *J*=14.6, 7.4, 5.0 Hz, 1H), 1.90 (ddt, *J*=14.5, 10.0, 7.2 Hz, 1H), 3.23 (dd, *J*=25.4, 1.2 Hz, 1H), 3.95 – 4.02 (m, 1H), 4.07 – 4.13 (m, 1H), 4.13 – 4.20 (m, 3H), 5.06 (dddd, *J*=12.3, 9.9, 5.1, 1.2 Hz, 1H), 7.34 (m, 1H), 7.35 – 7.38 (m, 1H), 7.51 (ddt, *J*=8.2, 6.8, 1.2 Hz, 1H), 7.69 – 7.73 (m, 1H), 7.88 (d, *J*=8.3 Hz, 1H), 8.50 (m, 1H). ^13^C NMR (Chloroform-*d*, 176 MHz): δ (ppm) 10.24, 16.18 (d, *J*=6.1 Hz), 16.38 (d, *J*=6.1 Hz), 26.59 (d, *J*=16.0 Hz), 52.21 (d, *J*=125.9 Hz), 63.01 (d, *J*=6.6 Hz), 63.43 (d, *J*=6.4 Hz), 78.29 (d, *J*=5.9 Hz), 113.43, 121.89, 124.86, 126.68, 128.48, 129.24, 129.39, 130.20, 138.38, 153.80, 187.35 (d, *J*=5.0 Hz). ESI-MS [M+H]^+^ = 363.1 Anal. Calcd for C_19_H_23_O_5_P: C, 62.98; H, 6.40;. Found: C, 63.11; H, 6.19.

Due to small amount of the enol form, the signals of enol form in the ^1^H and ^13^C NMR spectra have not been assigned.

Diethyl (2-butyl-4-oxo-3,4-dihydro-2*H*-benzo[*g*]chromen-3-yl)phosphonate (**13m**) (103.4 mg, 53%). White crystal, mp 92-96 °C. ^31^P NMR (Chloroform-*d*, 101 MHz): δ (ppm) 18.70 (cis), 20.27 (trans), 23.54 (enol). ^1^H NMR (Chloroform-*d*, 700 MHz): δ (ppm) 0.86 (t, *J*=7.3 Hz, 3H), 1.01 (t, *J*=7.1 Hz, 3H), 1.24 – 1.38 (m, 5H), 1.38 – 1.50 (m, 2H), 1.56 (ddt, *J*=14.6, 9.7, 5.6 Hz, 1H), 1.90 (dtd, *J*=14.6, 9.7, 5.1 Hz, 1H), 3.23 (dd, *J*=25.3, 1.1 Hz, 1H), 3.99 (m, 1H), 4.11 (m, 1H), 4.18 (m, 2H), 5.16 (dddd, *J*=12.1, 9.9, 4.9, 4.9, Hz, 1H), 7.35 (s, 1H), 7.38 (t, *J*=7.9 Hz, 1H), 7.53 (ddd, *J*=8.3, 6.7, 1.3 Hz, 1H), 7.72 (d, *J*=8.4 Hz, 1H), 7.89 (d, *J*=8.3 Hz, 1H), 8.53 (s, 1H). ^13^C NMR (Chloroform-*d*, 176 MHz): δ (ppm) 1.13, 13.97, 16.17 (d, *J*=6.0 Hz), 16.37 (d, *J*=6.0 Hz), 22.20, 27.86, 33.02 (d, *J*=15.7 Hz), 52.39 (d, *J*=125.4 Hz), 62.94 (d, *J*=6.7 Hz), 63.36 (d, *J*=6.4 Hz), 113.39, 121.88, 124.82, 126.67, 128.46, 129.21, 129.35, 130.19, 138.38, 153.86, 187.41 (d, *J*=5.0 Hz). ESI-MS [M+H]^+^ = 391.1. Anal. Calcd for C_21_H_27_O_5_P: C, 64.61; H, 6.97;. Found: C, 64.43; H, 7.07.

Due to small amount of the enol form, the signals of enol form in the ^1^H and ^13^C NMR spectra have not been assigned. Also, for the same reason, not all signals of cis form were assigned.

diethyl (2-isopropyl-4-oxo-3,4-dihydro-2*H*-benzo[*g*]chromen-3-yl)phosphonate (**13n**) (124.2 mg, 66%). White crystal, mp 112-116 °C.^31^P NMR (Chloroform-*d*, 101 MHz): δ (ppm) 18.13 (cis), 20.84 (trans), 24.05 (enol). ^1^H NMR (Chloroform-*d*, 700 MHz): δ (ppm) 0.93 (d, *J*=6.6 Hz, 3H), 0.97 (t, *J*=7.1 Hz, 3H), 1.03 (d, *J*=6.5 Hz, 3H), 1.32 (t, *J*=7.0 Hz, 4H), 1.95 – 2.05 (m, 1H), 3.44 (dd, *J*=25.9, 1.1 Hz, 1H), 3.94 (m, 1H), 4.05 – 4.18 (m, 3H), 4.67 (ddd, *J*=12.4, 10.3, 1.1 Hz, 1H), 7.33 (s, 1H), 7.34 (ddd, *J*=8.2, 6.8, 1.2 Hz, 1H), 7.49 (ddd, *J*=8.2, 6.8, 1.3 Hz, 1H), 7.69 (d, *J*=8.1 Hz, 1H), 7.86 (d, *J*=8.0 Hz, 1H), 8.48 (s, 1H). ^13^C NMR (Chloroform-*d*, 176 MHz): δ (ppm) 16.15 (d, *J*=6.0 Hz), 16.36 (d, *J*=6.2 Hz), 18.75, 19.25, 30.40 (d, *J*=14.9 Hz), 62.88 (d, *J*=6.8 Hz), 63.39 (d, *J*=6.7 Hz), 82.59 (d, *J*=6.1 Hz), 113.15, 122.28, 124.82, 126.67, 128.40, 129.07, 129.35, 130.17, 138.33, 154.14, 187.32 (d, *J*=4.8 Hz). ESI-MS [M+H]^+^ = 377.1. Anal. Calcd for C_20_H_25_O_5_P: C, 63.82; H, 6.70;. Found: C, 63.61; H, 6.49.

Due to small amount of the enol form, the signals of enol form in the ^1^H and ^13^C NMR spectra have not been assigned. Also, for the same reason, not all signals of cis form were assigned.

Diethyl (4-oxo-2-phenyl-3,4-dihydro-2*H*-benzo[*g*]chromen-3-yl)phosphonate (**13o**) (155.9 mg, 76%). White crystal, mp 74-178 °C.^31^P NMR (Chloroform-*d*, 101 MHz): δ (ppm) 16.80 (cis), 20.02 (trans), 22.82 (enol). ^1^H NMR (Chloroform-*d*, 700 MHz): δ (ppm) 0.84 (t, *J*=7.0 Hz, 3H, enol), 1.04 (t, *J*=7.1 Hz, 3H, trans), 1.34 (t, *J*=7.1 Hz, 3H, trans), 1.42 (t, *J*=7.0 Hz, 3H, enol), 3.42 (m, 1H, enol), 3.88 (dd, *J*=25.7, 1.7 Hz, 1H, trans), 4.05 (m, 1H, trans+enol), 4.13 – 4.25 (m, 3H, trans+enol), 5.88 (d, *J*=8.2 Hz, 1H, enol), 6.31 (d, *J*=12.2 Hz, 1H, trans), 7.09 (s, 1H, enol), 7.18 (d, *J*=7.3 Hz, 1H, trans), 7.22 – 7.26 (m, 1H, trans+enol), 7.30 – 7.33 (m, 1H, trans+enol), 7.34 (d, *J*=7.8 Hz, 1H, trans+enol), 7.38 (m, 1H, trans), 7.41 (s, 1H, trans), 7.48 (ddd, *J*=8.1, 6.7, 1.2 Hz, 1H, trans), 7.57 (d, *J*=8.2 Hz, 1H, enol), 7.69 (d, *J*=8.4 Hz, 1H, trans), 7.82 (m, 1H, trans+enol), 8.25 (s, 1H), 8.44 (s, 1H, enol), 11.41 (s, 1H, trans). ^13^C NMR (Chloroform-*d*, 176 MHz): δ (ppm) 15.55 (d, *J*=7.1 Hz, enol), 16.15 (d, *J*=6.0 Hz, trans), 16.33 (m, trans+enol), 51.87 (d, *J*=126.1 Hz, trans), 62.21 (d, *J*=4.1 Hz, enol), 62.58 (d, *J*=4.4 Hz, enol), 63.02 (d, *J*=6.8 Hz, trans), 63.54 (d, *J*=6.4 Hz, trans), 76.49 (d, *J*=14.7 Hz, enol), 77.43 (d, *J*=4.7 Hz, trans), 89.82 (d, *J*=184.2 Hz, enol), 112.36 (cis), 113.28 (trans), 122.15 (enol), 124.45 (enol), 124.86 (trans), 126.61 (trans), 126.66 (trans), 127.71 (trans), 127.83 (enol), 128.36 (trans) 128.44 (d, *J*=5.3 Hz, trans), 128.79 (trans), 129.02 (enol), 129.27 (trans), 129.37 (trans), 130.12 (trans), 136.21 (enol), 137.97 (d, *J*=16.0 Hz, trans), 138.25 (trans), 139.67 (enol), 151.85 , H, 154.52 , H, 161.17 , H, 186.83 (d, *J*=5.0 Hz, trans). ESI-MS [M+H]^+^ = 411.1. Anal. Calcd for C_23_H_23_O_5_P: C, 67.31; H, 5.65;. Found: C, 67.18; H, 5.41.

Due to small amount of the enol form, the signals of enol form in the ^1^H and ^13^C NMR spectra have not been assigned.

.

3. Copies of ^31^P, ^1^H and ^13^C NMR Spectra.

Diethyl (2-(2-hydroxyphenyl)-2-oxoethyl)phosphonate (**10a**)

Diethyl (2-(2-hydroxy-3-methylphenyl)-2-oxoethyl)phosphonate (**10b**)

Diethyl (2-(3-hydroxynaphthalen-2-yl)-2-oxoethyl)phosphonate (**10c**)

Diethyl (4-oxo-4*H*-chromen-3-yl)phosphonate (**12a**)

Diethyl (8-methyl-4-oxo-4*H*-chromen-3-yl)phosphonate (**12b**)

Diethyl (4-oxo-4*H*-benzo[*g*]chromen-3-yl)phosphonate (**12c**)

Diethyl (2-methyl-4-oxochroman-3-yl)phosphonate (**13a**)

Diethyl (2-ethyl-4-oxochroman-3-yl)phosphonate (**13b**)

Diethyl (2-butyl-4-oxochroman-3-yl)phosphonate (**13c**)

Diethyl (2-isopropyl-4-oxochroman-3-yl)phosphonate (**13d**)

Diethyl (4-oxo-2-phenylchroman-3-yl)phosphonate (**13e**)

Diethyl (2,8-dimethyl-4-oxochroman-3-yl)phosphonate (**13f**)

Diethyl (2-ethyl-8-methyl-4-oxochroman-3-yl)phosphonate (**13g**)

Diethyl (2-butyl-8-methyl-4-oxochroman-3-yl)phosphonate (**13h**)

Diethyl (2-isopropyl-8-methyl-4-oxochroman-3-yl)phosphonate (**13i**)

Diethyl (8-methyl-4-oxo-2-phenylchroman-3-yl)phosphonate (**13j**)

Diethyl (2-methyl-4-oxo-3,4-dihydro-2*H*-benzo[*g*]chromen-3-yl)phosphonate (**13k**)

diethyl (2-ethyl-4-oxo-3,4-dihydro-2*H*-benzo[*g*]chromen-3-yl)phosphonate (**13l**)

Diethyl (2-butyl-4-oxo-3,4-dihydro-2*H*-benzo[*g*]chromen-3-yl)phosphonate (**13m**)

Diethyl (2-isopropyl-4-oxo-3,4-dihydro-2*H*-benzo[*g*]chromen-3-yl)phosphonate (**13n**)

Diethyl (4-oxo-2-phenyl-3,4-dihydro-2*H*-benzo[*g*]chromen-3-yl)phosphonate (**13o**)

2-Methyl-3-methylenechroman-4-one (**14a**)

2-Ethyl-3-methylenechroman-4-one (**14b**)

2-Butyl-3-methylenechroman-4-one (**14c**)

2-Isopropyl-3-methylenechroman-4-one (**14d**)

3-Methylene-2-phenylchroman-4-one (**14e**)

2,8-Dimethyl-3-methylenechroman-4-one (**14f**)

2-Ethyl-8-methyl-3-methylenechroman-4-one (**14g**)

2-Butyl-8-methyl-3-methylenechroman-4-one (**14h**)

2-Isopropyl-8-methyl-3-methylenechroman-4-one (**14i**)

8-Methyl-3-methylene-2-phenylchroman-4-one (**14j**)

2-Methyl-3-methylene-2,3-dihydro-4*H*-benzo[*g*]chromen-4-one (**14k**)

2-Ethyl-3-methylene-2,3-dihydro-4*H*-benzo[*g*]chromen-4-one (**14l**)

2-Butyl-3-methylene-2,3-dihydro-4*H*-benzo[*g*]chromen-4-one (**14m**)

2-Isopropyl-3-methylene-2,3-dihydro-4*H*-benzo[*g*]chromen-4-one (**14n**)

3-Methylene-2-phenyl-2,3-dihydro-4*H*-benzo[*g*]chromen-4-one (**14o**)
